# Electronic structure study of YNbTiO$_6$ and CaNb$_2$O$_6$ with actinide impurities using compound-tunable embedding potential method

**DOI:** 10.1038/s41598-025-94297-3

**Published:** 2025-03-27

**Authors:** Daniil Maltsev, Yuriy Lomachuk, Vera Shakhova, Nikolai Mosyagin, Daria Kozina, Anatoly Titov

**Affiliations:** Petersburg Nuclear Physics Institute named by B.P. Konstantinov of National Research Center “Kurchatov Institute” (NRC “Kurchatov Institute” - PNPI), mkr. Orlova roscha, 1, Leningrad district, 188300 Gatchina, Russian Federation

**Keywords:** Computational chemistry, Density functional theory, Quantum chemistry

## Abstract

The compound-tunable embedding potential (CTEP) method is applied to study actinide substitutions in the niobate crystals YNbTiO$_6$ and CaNb$_2$O$_6$. Two one-center clusters are built and centered on Y and Ca, and 20 substitutions of Y and Ca with U, Np, Pu, Am, and Cm were made in four different oxidation states for each cluster. Geometry relaxation is performed for each resulting structure, and electronic properties are analyzed by evaluating the spin density distribution and chemical shifts of X-ray emission spectra. Though the studied embedded clusters with actinides having the same oxidation state are found in general to yield similar local structure distortions, for Am, Cm and Pu in high “starting” oxidation states the electron transfer from the environment was found, resulting in decrease of their oxidation states. The U substitutions are additionally studied with the use of multi-center models, which can provide both more structural and electronic relaxation and also include charge-compensating vacancies. For “starting” U$^\textrm{VI}$ case, the decrease in the oxidation state similar to that of Am$^\textrm{VI}$ and Cm$^\textrm{VI}$ in one-center clusters is observed in our calculations but in a different way, while for “starting” U$^\textrm{III}$ state the reverse process takes place, resulting in an increase in the oxidation state of uranium to U$^\textrm{IV}$. It is known experimentally that the Nb and Ti atoms in YNbTiO$_6$ are statistically distributed and occupy the same Wyckoff positions. With the CTEP method, it is possible to simulate to a certain extent the effects of such random distribution on the basis of perfect crystal calculation by performing Ti$\leftrightarrow$Nb substitutions in the embedded clusters. The results were compared to those obtained using the special quasirandom structures (SQS) method with structural relaxation for the single and double cell.

## Introduction

YNbTiO$_6$ and CaNb$_2$O$_6$ crystals are synthetic end-members of the euxenite-(Y) or polycrase-(Y), (Y,Ca,Ce,U,Th) (Ti,Nb,Ta)$_2$O$_6$ and fersmite, (Ca,Ce,Na)(Ti,Nb,Ta)$_2$(O,OH,F)$_6$, minerals correspondingly, which in turn are members of a wide euxenite group with general formula AB$_2$(O,OH,F)$_6$ (B = Ti,Nb,Ta; A can be a variety of metals). These minerals are known to contain rare earth and actinide atoms as natural impurities. Despite metamictisation due to radioactive impurities, minerals of the euxenite group were found to be considerably more resistant to both oxidation and leaching of uranium atoms than minerals of a closely related betafite/pyrochlore group (A$_2$B$_2$(O,OH,F)$_7$ and similar structures)^1–4^ and, therefore, they are considered promising matrices for long-term storage of high-level waste (HLW). In recent articles ^5,6^ the state of uranium isotopes in different oxidation states was studied in a natural metamict mineral of the polycrase group.

The other important area of application of YNbTiO$_6$ and CaNb$_2$O$_6$ is conditioned by their luminescence properties. YNbTiO$_6$ has self-activated luminescence^7^ while it can also serve as a host for rare-earth (RE) doped phosphors. Due to the equality of the oxidation states and similarity of ionic radii, Y$^{3+}$ $\rightarrow$RE$^{3+}$ substitution does not significantly distort the crystal and the complex luminescence mechanism can be tuned by controlling the selection of dopants and their concentrations^8–12^.

Both applications mentioned above are united by the impurities of *f* elements (lanthanides and actinides) that create a considerable challenge for computational methods to simulate properties of interest with an accuracy sufficiently high for a wide range of applications. Direct DFT calculations of *ab initio* periodic structure of compounds containing *f* elements with accuracy at the level of 0.1 eV and better for the energetic characteristics of the crystals (which corresponds already to the densities of electronic levels for one-electron excitations of localized and perturbed *d*, *f* states of *f* elements in compounds) are in general too demanding or challenging to date^13,14^. In turn, some program packages (e.g., plane wave codes vasp, pwscf, and abinit) utilize the Green-function-based GW-approximations for periodic structure studies, but their application to evaluating band gaps and electronic excitations at the above-mentioned accuracy is very expensive compared to DFT and usually requires expert art^15^.

For studying impurities in solids, the dynamical mean-field theory (DMFT)^16^, the density matrix embedding potential^17^ and other approaches^18^ are developed. To describe strongly correlated materials, which are of our main interest, one of the most popular approaches now is DMFT that is based on mapping a many-body lattice problem to a many-body local problem^19^ using the Green-function’s technique. However, note that these approaches are usually applied together with the DFT to structures containing *f*-elements.

However, to minimize computational efforts, various semi-empirical schemes are often used in practice for periodic structure calculations of compounds of *d*, *f*-elements and even for those containing heavy *p*-elements as impurities when utilizing some fitting parameters such as U in DFT + U studies^20^ to simulate exact exchange in the LDA and GGA functionals of DFT. Accurate studies of compounds with *f* elements often require considering spin-dependent relativistic effects, which complicate the crystallographic point group’s consideration^21^, but it can also be taken into account by using the above-mentioned codes. Some of the important examples here include transitions between the ferromagnetic and antiferromagnetic phases^22,23^.

Note that additional computational problems arise in calculations with periodic boundary conditions when a difference in oxidation state between the original and substitute atoms should be taken into account (i.e., the substitution charge differs from the original charge), which can result in the charge transfer effects and local reorganization of the fragment structure. Some other problems which cannot be accurately treated to date in general even within the supercell models of periodic structure studies include local breaking of the crystal symmetry by the impurities, static electron correlations which can be essential for *f* and *d* elements, partially occupied *f* and *d* shells spatially localized in the core region, etc. In contrast, precision at the level of 0.01 eV is now attainable for molecules which can contain even light actinides (e.g., see Figs. 7 and 8 in^24^ and^25^) when precise versions of relativistic pseudopotentials and coupled cluster methods are applied (see discussion in the Introduction of^26^).

Returning to the structures of our interest in the given research, one more problem should be discussed here: Last but not least, the Nb and Ti atoms are statistically distributed in the practically synthesized YNbTiO$_6$ “crystals” (see^27^). There exist several methods that are commonly used to treat such systems. These methods are briefly discussed below.

The virtual crystal approximation (VCA)^28^ and the coherent potential approximation (CPA)^29^ are based on the simulation of an average effective crystal. In VCA, the statistically distributed atoms are replaced by the fictitious “virtual” atoms with interpolated potentials (for example, by constructing a linear combination of atomic pseudopotentials). In the CPA method, the ensemble-averaged one-particle Green’s function is used. Although these approaches are commonly used because they are rather universal, they cannot be applied to accurate studies of such structures (known also as high-entropy alloys, HEAs) and do not describe different local atomic environments due to the statistically distributed atom, including lattice distortions with breaking the translational symmetries, etc.^30^.

In the cluster expansion (CE) method^31,32^ the scalar properties of the crystal are derived from all individual configurations (particular arrangements of statistically distributed atoms on the sublattice) and each configuration is described in terms of “interaction” contributions of clusters, where the clusters consist of one or more lattice sites. The crucial step in the CE method is to assume that short-range interactions are more important than long-range ones, and therefore to neglect the contributions from all clusters with atoms located at large distances.

The Special Quasirandom Structures approach (SQS)^33,34^ is based on the CE method; however, instead of considering all possible configurations, the single configuration (cell or supercell) is selected so that its correlation functions are most close to those of the perfectly disordered structure. The use of a single configuration drastically reduces computational cost compared to CE. However, postulating the perfect disordered structure as a desirable goal leads to systematic errors when the short-range order is significant^35^. Being initially developed for metal alloys and semiconductors, the SQS method was also applied to complex oxides, including niobate crystals from the pyrochlore and perovskite groups^36–39^.

Although all of these methods can be applied to obtain bulk properties of a disordered crystal, the task of simulating local properties and point defects remains a complex one. The VCA and CPA methods do not allow one to perform independent local relaxations of individual sites due to their “averaged” nature and, in particular, do not include the specific nonlinear effects characteristic for the individual atoms. The CE method with the local relaxation taken into account requires significant computation efforts, which are additionally (and dramatically) increased with the size of the unit cell and basis set. The latter considerably limits the application area of the CE method. The selected configuration of the SQS approach contains a somewhat representative set of local configurations; however, to study local properties and point defects, the same problems that were discussed for a perfect crystal but multiplied by the number of individual sites still need to be solved.

One of the most reasonable ways to overcome these problems is to apply the embedding potential theory for a crystal fragment study, which makes it possible to simulate effective crystal surroundings by some operator-type potential acting from the environment onto the fragment and, thus, resulting in calculations of a limited-size cluster of molecular type. This technique allows one to use much more advanced methods and computational parameters than those in conventional periodic-structure calculations. This approach allows one to simulate local perturbations including point defects, such as substitutions. Additionally, the rearrangement of statistically distributed atoms can be estimated to some extent by making substitutions or modifying the embedding parameters (for example, Nb and Ti in YNbTiO$_6$).

The idea of applying embedding potential theory (see^18,40–42^ and references) to describe fragments of a crystal without considering the whole periodic structure of the crystal has a rather long history. Mention here only some of the milestone developments such as generalized product functions^43^, theories of electron separability^44^, and Adams-Gilbert formalism^45^, which resulted in the Huzinaga-type^44,46,47^ or *ab initio* model potential (AIMP) in their latest developments; see^48,49^ for molecular calculations. Some first AIMP applications for atomic ion defects are made in^50,51^, and the use of the AIMP version of the embedding fragment in doped solids is given in^52^. Concerning the latest applications of the AIMP embedding theory, mention the study of Joos with co-workers^53^ devoted to the charge transfer from Eu$^{2+}$ to trivalent lanthanide co-dopants in the host CaF$_2$ crystal and the paper of Larsson and Veryazov^54^ devoted to the CASPT2 study of the Ce:YVO$_4$ spectrum.

An advantage of the AIMP approach is the possibility of approximate freezing (see below) of nonspherical (valence) orbitals localized on environmental atoms that can be important if these atoms are closely located to the crystal fragment under consideration^55^. However, in constructing the nonspherical crystalline orbitals to be used as “frozen” in the level-shift AIMP projectors, one should rather apply some localizing projectors for these orbitals, which, in turn, can be correctly constructed in some *ab initio* scheme only after a preliminary periodic structure calculation of the compound. However, this can be a complicated procedure, and in practice (see^54^ and references), some empirical and model assumptions for both the equilibrium geometry of the crystal and the above localized orbitals are used to simplify such calculations dramatically within the AIMP method.

In addition, for studying heavy-atom systems one should use relativistic AIMP versions, which are reviewed in^56^. They first require some two-component all-electron approximation (one-component versions for scalar-relativistic applications) for the effective Hamiltonian and wave functions to use AIMP in practical applications. In^57^ the combined use of the AIMP technique together with shape-consistent relativistic effective core potentials^58–60^ (pseudopotentials or PPs for brevity below) was proposed. (Note that the “norm-conserving” PP formulations^61–63^ are mainly used in periodic structure calculations, which are very close in essence to the shape-consistent PPs). However, it was shown in papers^64–66^ that the typical AIMP level-shift parameter $+2|\epsilon _i|$ (*i* numerates frozen orbitals and $\epsilon _i$ are their typically negative orbital energies; see Introduction in^67^ for more details) is too small by amplitude to achieve high accuracy of “freezing” the corresponding orbitals in the cases of accurate WFT methods (when electronic correlations are considered explicitly). In the above papers, the level shift parameter was at least one order of magnitude higher, to attain the uncertainties at the level of $0.03\div 0.01$ eV for the valence (chemical or spectroscopic) energies that is critical for *f*- and heavy *d*-elements having typical valence energies ${\sim }0.1$ eV and smaller.

As an alternative way to simulate the environment within the framework of the embedded cluster model, mention some first application of a semilocal shape-consistent PP method^58^ to study Cu$^+$ ion impurity in a NaF host crystal^68^ that was done soon after the large-core PP^69^ for Na and other atoms (ions) were generated. Note that the long-range interaction of the crystal fragment with the environment was simulated by several approximations to the lattice potential in the region of the cluster, which were compared to the “exact” Madelung potential. However, as discussed in^70^, the use of “universal” semilocal large-core PPs and Madelung-type potential to describe environmental atoms can hardly provide accuracy at the level of 0.1 eV for valence energies.

In the recently developed compound-tunable embedding potential (CTEP) method^26,70–72^ (see the next section for details), the large-core semilocal (or radially-local) PPs are generated by such a way that they are not universal but “tuned” to a particular crystal under consideration. Then, the point charges of the atoms in a near environment are tuned to reproduce the Coulomb potential of the environment within the considered fragment. As a result, the inherent accuracy of CTEP (in reproducing the fragment in its cluster consideration with CTEP compared to that in periodic structure calculations) can be as high as is generally required to describe compounds of *f*- and heavy *d*-elements (the errors can be much less than 0.1 eV for energies of valence electrons of the atoms from the fragment).

CTEP method was already applied to a CaNb$_2$O$_6$ crystal, which is the end member of the fersmite mineral belonging to the same euxenite group^26^; its precision was estimated by comparing the electronic densities in the embedded clusters with that of the original crystal. The effects of the increase in the basis set size were tested and two ways of simulating Ca$\rightarrow$U substitutions were compared.

In the present study, the CTEP method is applied to YNbTiO$_6$ and CaNb$_2$O$_6$ crystals. Note that the pilot application of CTEP in^26^ was mainly aimed at estimating the applicability of the method to niobate crystals, and only Ca$^{2+}$ $\rightarrow$U$^{4+}$ and Ca$^{2+}$ $\rightarrow$U$^{6+}$ substitutions were briefly studied there. Here, actinide substitutions (A$\rightarrow$M$^{n+}$; A=Y$^{3+}$,Ca$^{2+}$; M = U, Np, Pu, Am, and Cm; n = 3,4,5,6) are modeled for both crystals, and the resulting structures are thoroughly studied. Although comparison of different actinide substitutions is made with the use of the single-center cluster model, a larger three-center cluster is used for a more detailed study of uranium defects. Being more computationally challenging, such an extended model can take into account more structural and electronic relaxation. Moreover, since most actinide substitutions under study are charged crystal defects, the multi-center model is beneficial in that it allows inclusion of additional charge-compensating defects, such as vacancies.

## CTEP method

The detailed description of the CTEP method is given in our papers^26,70–72^. The general idea of CTEP is to select some crystal fragment and simulate the influence of the crystal environment by the CTEP operator, which is presented in the form of a linear combination of specific short-range semilocal pseudopotentials (see^24,57,73–76^ and references) for the atoms of the nearest environment and long-range Coulomb potentials (the action of which is taken into account only within the fragment) from optimized fractional point charges centered on both nearest and some more distant atoms of the environment, in general.

The crystal fragment is not restricted to having any symmetry corresponding to the crystal space group; however, it is considered as a set of alternating anionic and cationic “coordination spheres” around one or more corresponding cationic or anionic centers. In short, four steps are necessary to generate a CTEP for a crystal fragment of interest (cluster below):

(1) High-quality periodic-structure DFT calculation of the perfect crystal with geometry optimization. In general, medium-core PPs ^70^ with corresponding basis sets are used for heavy cations (particularly, for $d,f-$elements) and anions, while all-electron basis sets can be used for light anions. The basis sets used here are as good as possible to avoid the basis set linear dependence and other computational problems.

(2) Generation of large-core “compound-tunable” pseudopotentials (lc-CTPP) for all cations. The initial approximation for lc-CTPP is prepared as a large-core pseudopotential built for the effective state of the original atom in the crystal, with this state being preliminarily obtained by one of the population analysis methods (note that lc-CTPPs for nonequivalent (at different Wyckoff positions) atoms of the same type are generated independently). Next, lc-CTPPs are optimized by variation of a selected set of parameters (i.e. effective radius / exponent or coefficients of primitive gaussians) with the criterion of minimization of the root mean square (RMS) value of energy gradients with respect to coordinates of atomic nuclei for a crystal with the original medium-core PP replaced by the generated lc-CTPP. The basis sets for the lc-CTPPs are generated from the original basis sets for the corresponding atoms in the crystal by tuning the core part in order to match the behavior of the lc-CTPP pseudofunctions. When the lc-CTPPs are optimized, they are applied to any embedded cluster built for the given compound.

(3) Building a cluster from several parts: (a) “main cluster”, which is equal to or contains a crystal fragment of interest and must consist of one or more cationic centers and all their direct anionic neighbors; (b) nearest cationic environment (NCE) that contains the cations neighboring the main cluster; and (c) nearest anionic environment (NAE), which contains the anions neighboring the NCE except the main-cluster anions. Such a generated environment is sufficient for our clusters; however, note that for the main clusters with more complicated or oblong structures, the number of NCE and NAE spheres can be twice as many. The main-cluster atoms are initially treated by the same exchange-correlation functionals, pseudopotentials, and basis sets as in the periodic-structure calculation; NCE is represented by “pseudoatoms”, modeled by lc-CTPPs and reduced basis sets combined with partial point charges; and NAE are represented by negative partial point charges only (however, in some cases, simplified PPs can be added to the NCE pseudoatoms). All of the atoms and pseudoatoms are located at the theoretically optimized lattice sites of corresponding atoms in the original crystal to provide correct reproducibility of the main-cluster electronic structure when generating CTEP in addition to the constraints mentioned above on the PPs and basis sets. However, in some cases, rather arbitrary point charges outside the main cluster can also be added, in principle, to reproduce a long-range electrostatic potential.

Initial distribution of the partial charges is obtained by solving a system of linear equations for the charge transfer:

$$\begin{aligned} \sum _{j}^{j \in \textrm{neighbors}(i)}\textrm{CT}_{i\rightarrow j}=\textrm{RedOx}_{i}\ , \end{aligned}$$

where $\textrm{CT}_{i\rightarrow j}$ is an estimated formal charge transfer from atom *i* to *j* ($\textrm{CT}_{j\rightarrow i}=-\textrm{CT}_{i\rightarrow j}$), and $\textrm{RedOx}_{i}$ is the oxidation state of the corresponding atom in the crystal. The system is usually an underdetermined one, so a minimum norm solution is used.

When $\textrm{CT}_{i\rightarrow j}$ are found, the partial charges are estimated as

$$\begin{aligned} Q_{i \in \mathrm NCE} = \textrm{RedOx}_{i}\ , \end{aligned}$$

and

$$\begin{aligned} Q_{i \in \mathrm NAE} = \sum _{j}^{j \in \mathrm NCE}\textrm{CT}_{i\rightarrow j}\ . \end{aligned}$$

(4) “Optimization” of NCE and NAE point charges, made by variation of the charges within certain limits (usually from zero to the oxidation number) with the criterion of minimization of RMS forces on the nuclei of the main cluster.

After these steps are successfully completed, the preparation of the embedding potential is finished. The resulting cluster with CTEP can be used in various ways. The accuracy of the calculation can be increased by applying one of the advanced wave function-based correlation methods^72^ instead of DFT or by increasing the basis set and / or using small core PPs. Different atom-in-compound (AiC) properties on heavy atoms (see detailed discussion about it in papers^77–82^) can be studied within the two-step approach (see details in papers^83–87^). The diversity of structural and electronic perturbations can be considered in the main cluster, and point defects can be simulated, where one or multiple centers are replaced by the other atom or vacancy (taking into account that the positions and charges of NCE and NAE are fixed for all perturbations and replacements within the basic CTEP scheme discussed here).

## Computational details

The crystal code^88^ with the unrestricted hybrid functional PBE0^89^ was used to perform electronic structure calculations of crystals with periodic boundary conditions. A slightly modified so-dft code from the nwchem package^90^ was used for unrestricted PBE0 DFT calculations of the clusters.

The basis sets and PPs for the metal atoms generated by our group ^76^ were also used. The basis set generation procedure was slightly improved compared to our previous study ^26^, so that both periodic-structure and cluster calculations of CaNb$_2$O$_6$ were performed again. All PPs and basis sets used for the periodic-structure calculations and single-center CTEP clusters are presented in the supplementary material.

## Results and discussions

### Periodic structure calculation of YTiNbO$_6$

**Figure 1:**
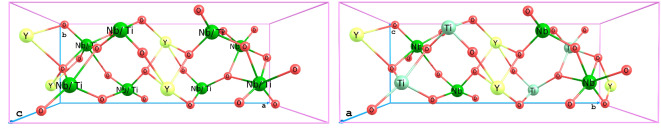
Statistically-averaged experimental (left) and calculated (right) conventional cells for YNbTiO$_6$.

In the synthesized crystals, in practice, the Ti and Nb atoms in YTiNbO$_6$ are statistically distributed, occupying the same position, each with probability 50%. Such a structure cannot be calculated directly, so a model crystal was constructed. Although the quasirandom cell structures are usually considered to be a better approximation to a disordered crystal, we chose a high symmetry cell in which all atoms of Y, Nb, and Ti are respectively equivalent to avoid ambiguity. Furthermore, the cell chosen in the experimental geometry (Fig. 1) had the lowest energy of all possible permutation configurations. However, the symmetry of the chosen cell was reduced from the original Pbcn (60) space group to P2$_1$/c (14) with only two of the cell angles remaining right.

The calculated and experimental parameters are compared in Table 1. Overall agreement is satisfactory, considering the inevitable distortions of YNbTiO$_6$ resulting from the splitting of the Ti and Nb positions and the decrease of the symmetry. It is worth noting that the Y-O distances in YNbTiO$_6$ are reproduced with high precision, while there are significant differences for the Nb-O and Ti-O distances.

**Table 1:** Experimental and calculated structural parameters for CaNb$_2$O$_6$ and YNbTiO$_6$. The cell vector order differs for YNbTiO$_6$ due to the change of the space group in the calculated structure.

Parameter	CaNb$_2$O$_6$			YNbTiO$_6$	
	Exp.^91^	Prev.^26^	Calc.	Exp.^27^	Calc.
a, Å	14.926	14.920	15.006	14.643	5.166
b, Å	5.752	5.690	5.747	5.553	14.649
c, Å	5.204	5.194	5.210	5.195	5.543
V, Å$^3$	446.8	440.9	449.4	422.4	419.4
$\alpha$	90$^{\circ }$	90$^{\circ }$	90$^{\circ }$	90$^{\circ }$	90$^{\circ }$
$\beta$	90$^{\circ }$	90$^{\circ }$	90$^{\circ }$	90$^{\circ }$	89.45$^{\circ }$
$\gamma$	90$^{\circ }$	90$^{\circ }$	90$^{\circ }$	90$^{\circ }$	90$^{\circ }$
Ca-O, Å	2.32–2.80	2.33–2.71	2.36–2.76	–	–
Y-O, Å	–	–	–	2.30–2.50	2.27–2.53
Ti-O, Å	–	–	–	1.72–2.41	1.83–2.24
Nb-O, Å	1.77–2.34	1.78–2.35	1.78–2.37	1.72–2.41	1.76–2.30

### One-center clusters for perfect CaNb$_2$O$_6$ and YNbTiO$_6$ crystals

**Figure 2:**
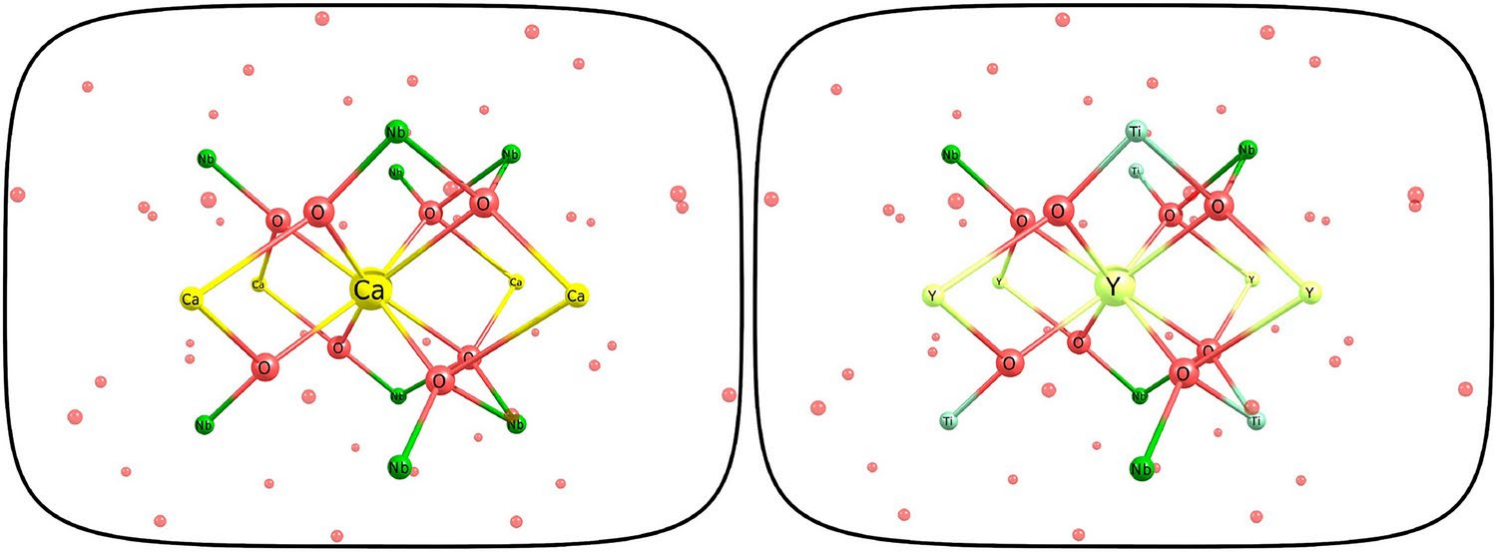
Ca-centered main cluster in CaNb$_2$O$_6$ (left) and Y-centered main cluster in YNbTiO$_6$ (right).

The crystals of the euxenite group have the general formula AB$_2$X$_6$, where $f-$elements usually replace atoms of the A group. One-center clusters with AO$_8$@CTEP formula for CaNb$_2$O$_6$ (A=Ca) and YNbTiO$_6$ (A = Y) are presented in Fig. 2. Structures and partial charges for both clusters are presented in the Supplementary Materials.

The agreement with the perfect crystal geometry for the Y-centered main cluster in YNbTiO$_6$ was found to be on the same level as that of the Ca-centered main cluster in CaNb$_2$O$_6$ (Table 2).

**Table 2:** Forces on the atoms of the main cluster and atomic displacements within the main cluster after its optimizations.

Structure	RMS force (a.u.)	RMS displacement(Å)
Ca_c_ (CaNb_2_O_6_)	1.2 · 10^-5^	4.4 · 10^-5^
Y_c_ (YNbTiO_6_)	1.9 · 10^-5^	1.9 · 10^-4^

### Basis set increase effects

After the cluster is successfully built, it is possible to modify the calculation parameters to achieve specific goals. One of the simplest modifications is the increase of the basis sets on one or several atoms and/or pseudoatoms, which allows one to answer two questions: (1) how stable is the main cluster geometry towards the variation of the basis sets and (2) can the basis set increase lead to more accurate geometry (closer to the experimental one). However, the latter test is hampered by the fact that the embedding potential is built for non-experimental geometry, which means that CTEP rather mitigates only a part of the deviations of the discussed theoretical model from the experiment. Additionally, for YNbTiO$_6$ there is a specific inevitable source of geometry errors arising from the lower symmetry of the perfect crystal used in the calculations, compared to a statistically averaged one, corresponding to the spectroscopic data (equivalent to the CaNb$_2$O$_6$ crystal symmetry).

**Figure 3:**
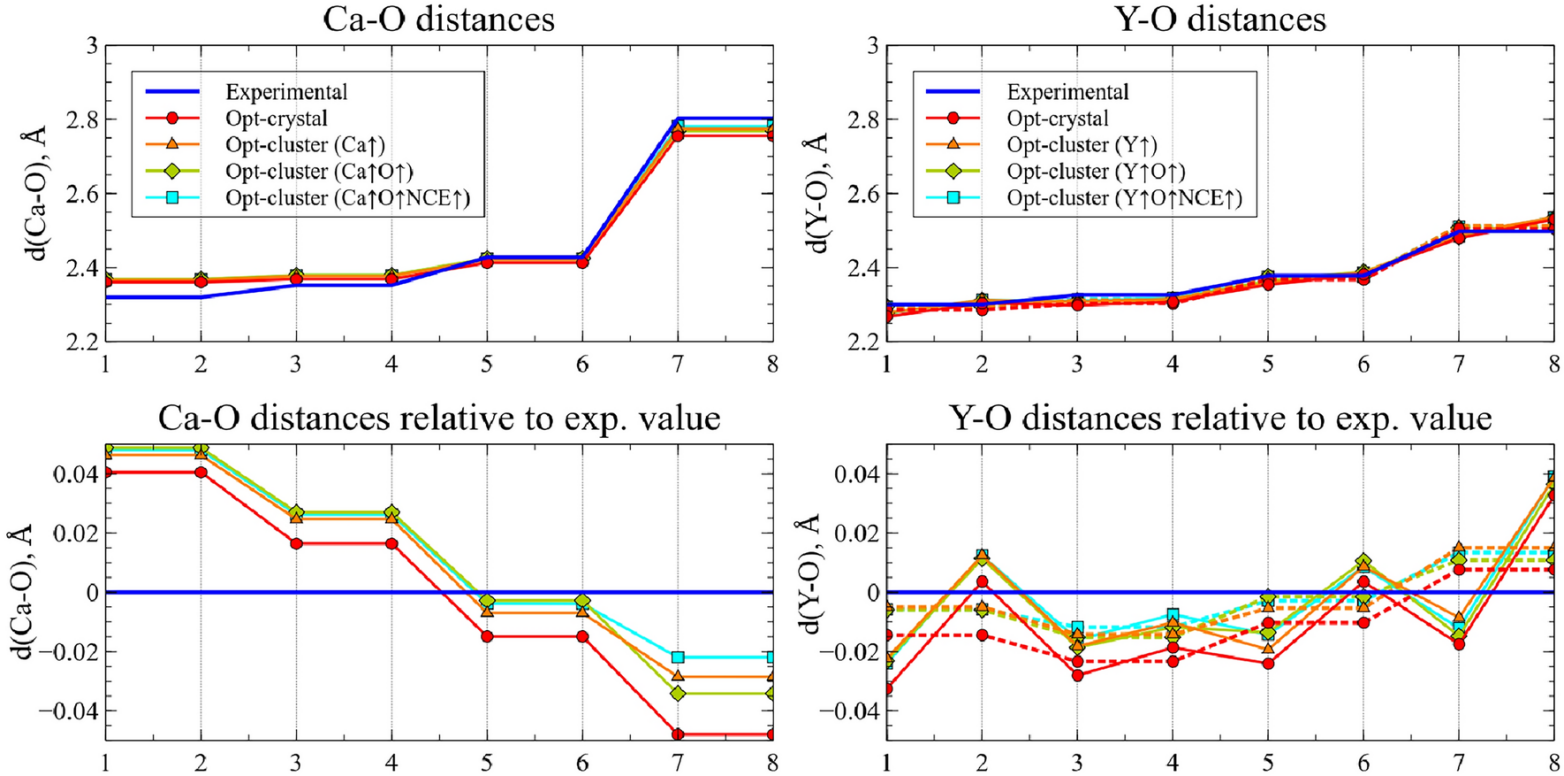
Ca-O and Y-O distances in the one-center main cluster for different basis sets. Numbers on x axis represent index of each O neighbor of the central atom. “Opt-crystal” corresponds to both periodic optimized structure and cluster with the same basis sets. Up arrows denote increased basis for the corresponding atoms. For YNbTiO$_6$ clusters real (solid lines) and averaged (dashed lines) Y-O distances are shown. The averaging is made for pairs of O atoms located on equal distances from Y atom in the experimental structure.

Several modifications to the basis set were made: Starting with the original parameters of the crystal calculations, the basis sets were successively increased in the valence region for (1) the central atom (Ca for CaNb$_2$O$_6$ and Y for YNbTiO$_6$; (2) its O neighbors; and (3) NCE pseudoatoms (see the supplementary materials for all the basis sets involved). The results obtained are presented in Fig. 3 and compared with the experimental data. The Y-O distances in the experimental structure of YNbTiO$_6$ are grouped into pairs due to local symmetry, so, for ease of comparison, each cluster for YNbTiO$_6$ corresponds to two graphs: the real geometry (solid line) and distances averaged by pairs (dashed line). In addition, to make it less cluttered, differences from the experimental values are shown in the bottom graphs.

The graph shows opposite tendencies for CaNb$_2$O$_6$ and YNbTiO$_6$: the smallest distances are overestimated and the largest are underestimated for the former, while the smallest distances are usually underestimated and the largest are usually overestimated for the latter. However, in both cases, the tendency can be explained by the idea that while the six closest neighbor oxygen atoms can be considered as direct chemically bound neighbors, the farthest two are loosely bound “semi-neighbors” and the corresponding distances are determined by the closest ones. So, the shortest distances to the closer neighbors lead to a stronger pushing out of the farthest ones, and vice versa. The increase of basis sets, in general, leads to the increase of all the distances.

In general, there is fairly close agreement between the experimental geometries and the calculated data (although it was not completely expected for YNbTiO$_6$ due to differences between a real disordered crystal and the chosen perfect one). It is also important that the relaxations resulting from the basis-set expansion are in both cases small and seem to be converging, which allows us to assume that the chosen basis set is full enough for our goals.

### Actinide substitutions

Both clusters, which simulate the fragments of calcium niobate and perfect yttrium titanoniobate crystals, were used to model the substitution A$\rightarrow$ M$^{n+}$ (A = Ca$^{2+}$ for CaNb$_2$O$_6$ and Y$^{3+}$ for YNbTiO$_6$; M = U, Np, Pu, Am, Cm; n = 3, 4, 5, 6), with a total of 40 actinide-substituted clusters were constructed The structural relaxation (with NCE and NAE pseudoatoms being fixed) was performed and the distribution of the spin density was calculated for each substituted cluster.

**Figure 4:**
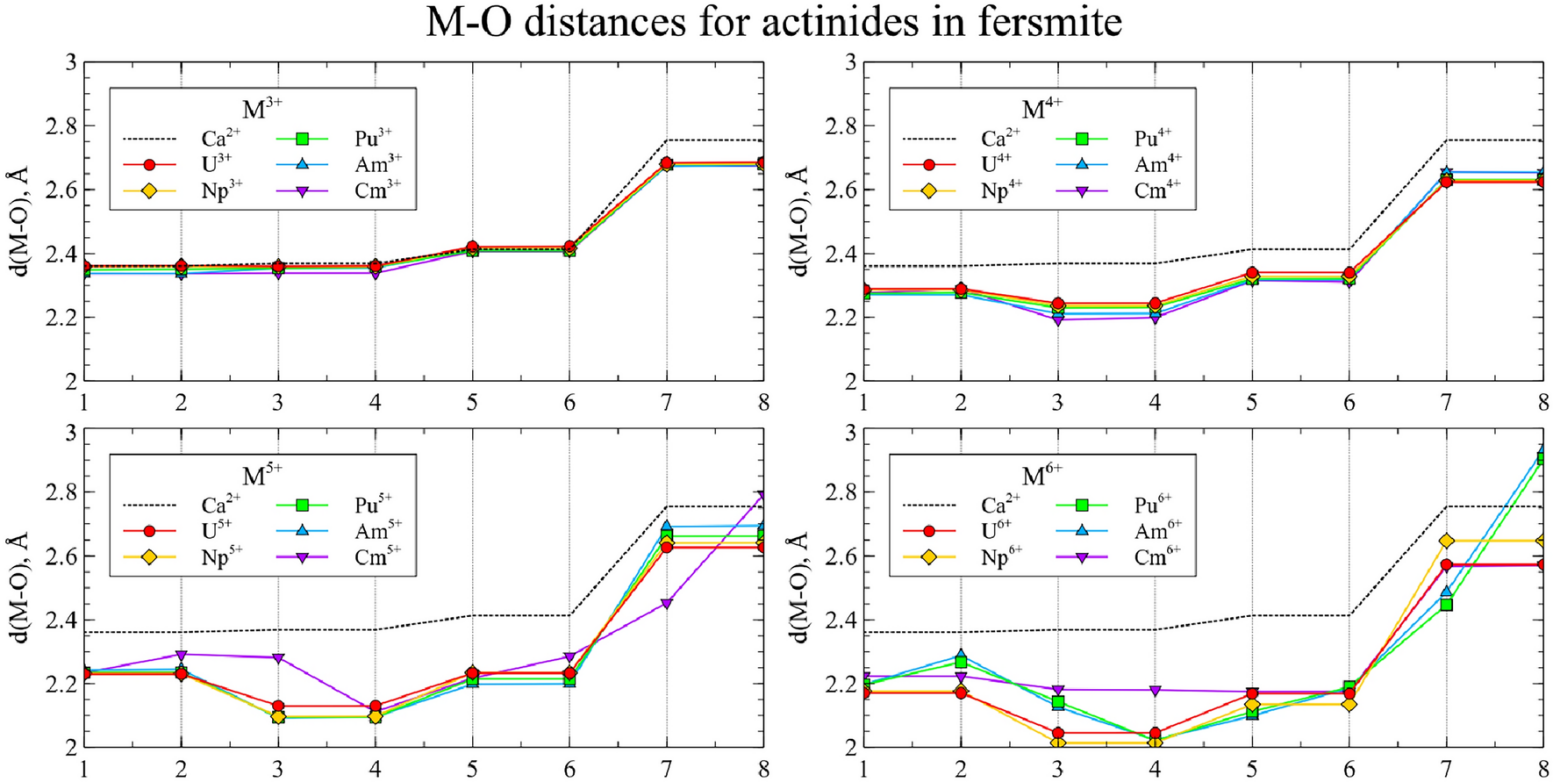
The M-O distances for actinide substitutions in CaNb$_2$O$_6$. Numbers on the *x* axis represent index of each O neighbor of the central atom. Dashed line corresponds to Ca-O distances in the perfect crystal.

**Figure 5:**
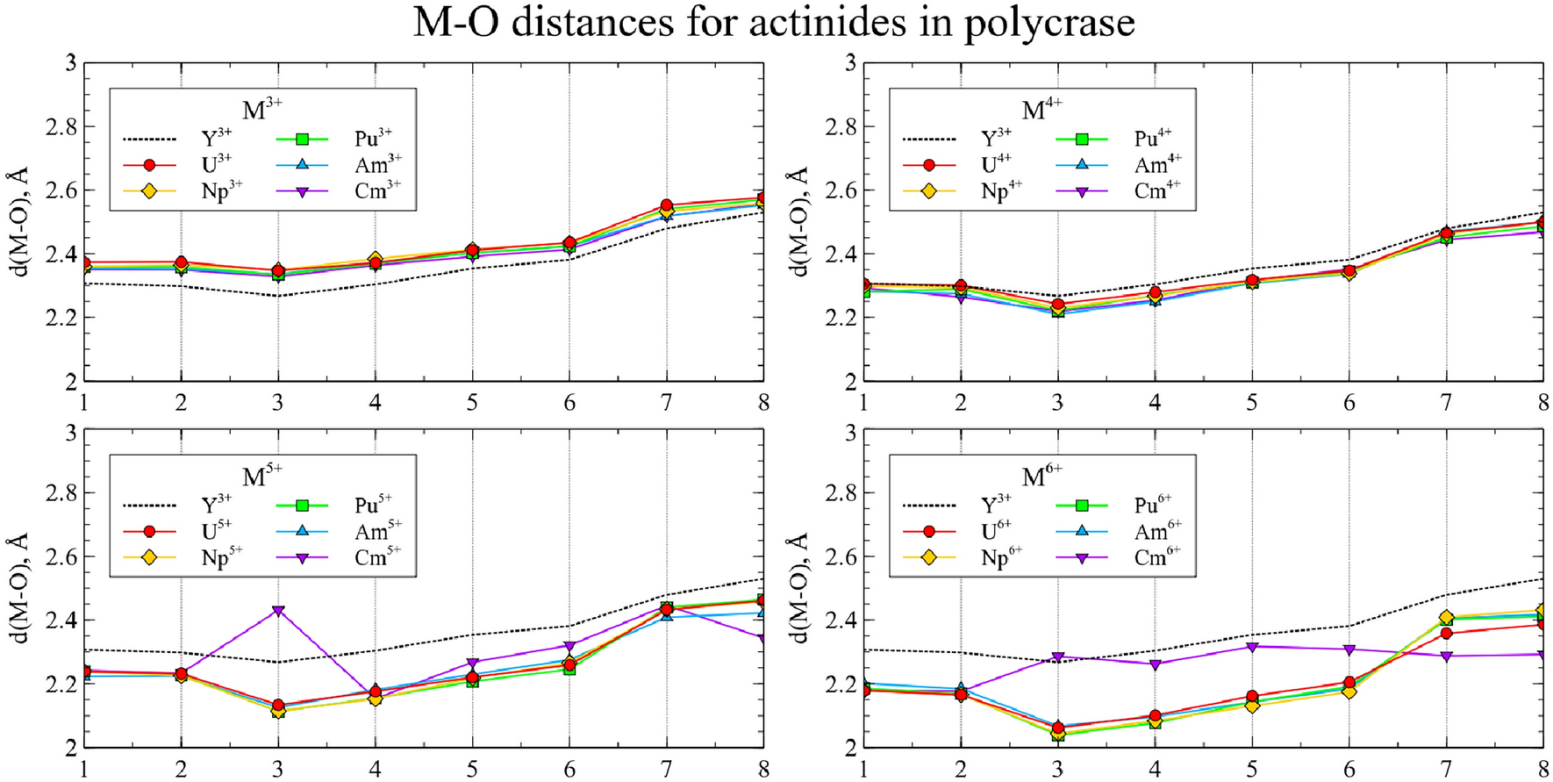
The M-O distances for actinide substitutions in YNbTiO$_6$. Numbers on the *x* axis represent index of each O neighbor of the central atom. Dashed line corresponds to Y-O distances in the perfect crystal.

In Figs. 4 and 5 the relaxed main cluster structures are presented as a set of M-O distances.

Actinides in the same oxidation state, as a rule, yield similar structures. Thus, one can assume that such substitutions will exhibit similar physical and chemical properties, and data for uranium can be extrapolated to the rest of the actinides under study. However, several actinides in high oxidation states demonstrate exceptions to this rule. To investigate these deviations and obtain more detailed data, the spin densities were calculated for each cluster and integrated as a function of distance to the central atom:

$$\begin{aligned} sd(r) = \frac{1}{4\pi }\oint d \Omega \left| \rho _{spin}(\vec {r}) \right| \end{aligned}$$

**Figure 6:**
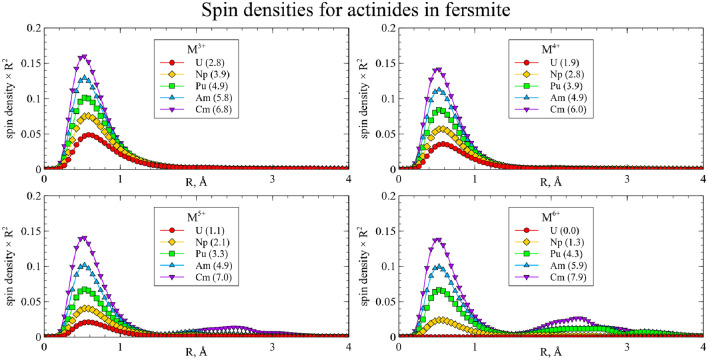
Integrated spin densities for actinide substitutions in CaNb$_2$O$_6$. Total values of integrated spin density are given in parentheses in the legend.

**Figure 7:**
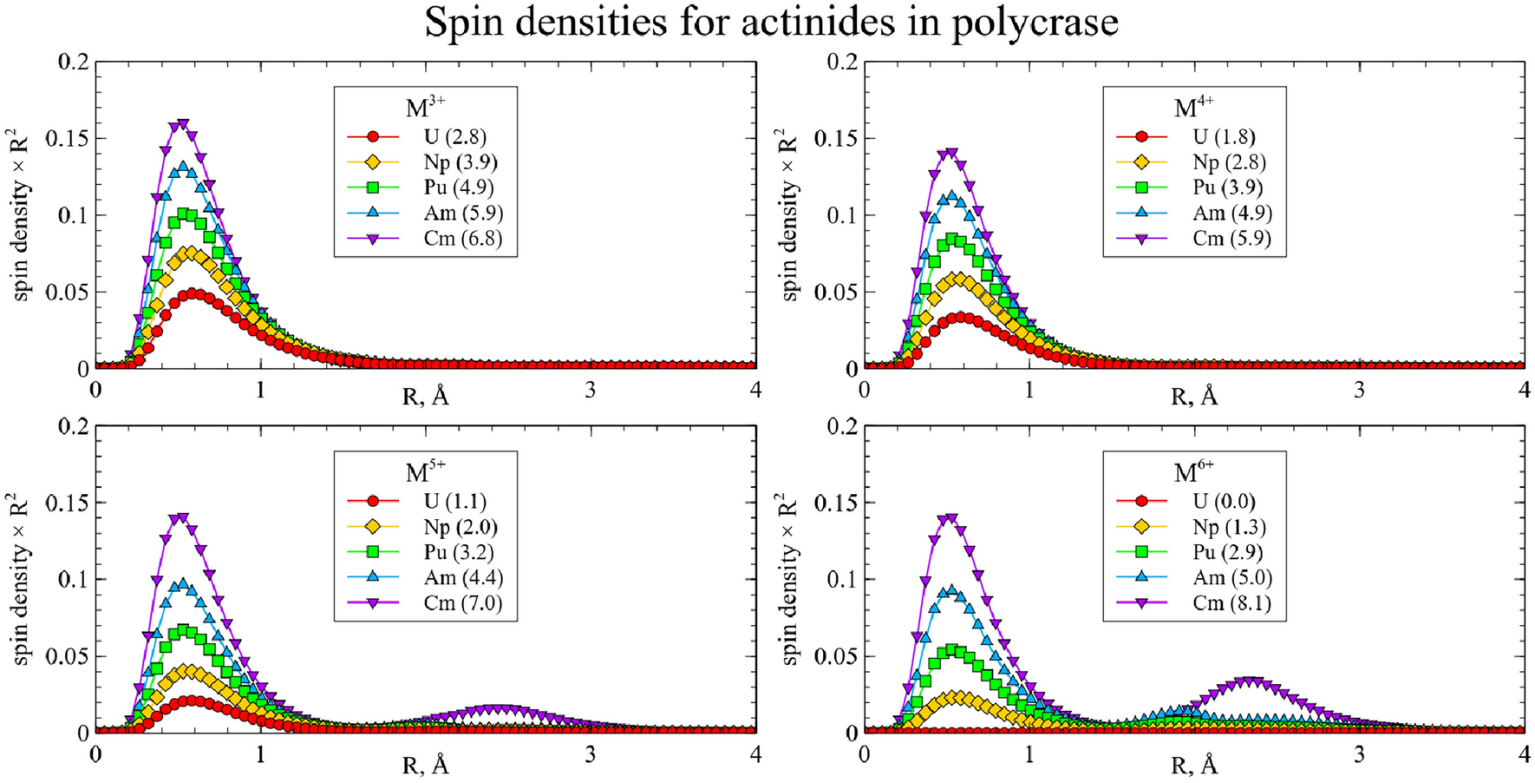
Integrated spin densities for actinide substitutions in YNbTiO$_6$. Total values of integrated spin density are given in parentheses in the legend.

The resulting graphs are shown on Figs. 6 and 7. The maximum spin density, associated with the radii of *f* orbitals, is almost equal for all clusters. For most of the clusters, the spin density is localized on the actinide atom, and the number of unpaired electrons increases with increasing atomic number and decreases with increasing oxidation state.

However, there are several deviations where increase in the formal oxidation state does not result in decrease of the spin density on the central atom but instead the secondary spin density peaks appear at distances close to those of the neighboring oxygen atoms. This indicates the electron transfer from the one or several oxygen atoms to the metal center, so that the resulting effective oxidation state is lower than the preassigned one. For CaNb$_2$O$_6$ this happens for Cm$^{5+}$, Cm$^{6+}$, Am$^{6+}$ and Pu$^{6+}$, while for YNbTiO$_6$ it happens for Cm$^{5+}$, Cm$^{6+}$ and Am$^{6+}$. Furthermore, partial electron transfer occurs for Am$^{5+}$ in CaNb$_2$O$_6$ and for Pu$^{6+}$ in YNbTiO$_6$. Although such results can be explained by the inaccuracy of the applied model, the different interpretation is that the obtained results can serve as an indication that the mentioned oxidation states of the actinides as substituents cannot exist in the considered fragments of niobate matrices in practice. The circumstance that the set of unachievable oxidation states is wider for CaNb$_2$O$_6$ than for YNbTiO$_6$ must correspond to lower charge difference for Y$^{3+}$ $\rightarrow$M$^{n+}$ substitutions than for Ca$^{2+}$ $\rightarrow$M$^{n+}$ ones: higher charge difference is likely to make the corresponding cluster less stable to electronic relaxation. When problematic oxidation states are excluded, the remaining ones have very close structural properties for the same oxidation states.

### X-ray line chemical shifts

Chemical shifts of the lines of X-ray emission spectra (chemshifts) for the actinides were calculated for all substitutions under study. Our results are collected in Table 3.

**Table 3:** X-ray line chemical shifts (meV) for actinides in CaNb$_2$O$_6$ and YNbTiO$_6$ relative to free M$^{4+}$ ion.

	CaNb$_2$O$_6$		YNbTiO$_6$	
	K$\alpha _2$	K$\alpha _1$	K$\alpha _2$	K$\alpha _1$
U$^{3+}$	− 129.2	− 165.4	− 123	− 157.6
U$^{4+}$	39.1	59.3	16.7	22.6
U$^{5+}$	167.3	221.8	141.9	185.6
U$^{6+}$	246.5	324.9	223.6	292.5
Np$^{3+}$	− 189.1	− 241.4	− 181.4	− 231.6
Np$^{4+}$	− 6	− 7.9	− 0.9	− 1.4
Np$^{5+}$	125.1	160.5	137.6	176.6
Np$^{6+}$	209.5	267.8	222.3	284.4
Pu$^{3+}$	− 191.9	− 250.9	− 185.4	− 242.7
Pu$^{4+}$	− 8.6	− 16.1	− 3.1	− 8.7
Pu$^{5+}$	101.7	126	115.7	144.5
Pu$^{6+}$	[112.7]	[140.1]	174.7	219.3
Am$^{3+}$	− 178.1	− 231.8	− 173.1	− 223.7
Am$^{4+}$	− 15.8	− 24.2	− 9.1	− 15.5
Am$^{5+}$	55.4	66.9	82.7	102
Am$^{6+}$	[66.9]	[81.1]	[101.9]	[124.9]
Cm$^{3+}$	− 197.9	− 255	− 194.6	− 250.3
Cm$^{4+}$	− 37	− 48.4	− 25.7	− 32.9
Cm$^{5+}$	[− 20.9]	[− 26.7]	[− 22.6]	[− 28.6]
Cm$^{6+}$	[− 3.1]	[− 4.1]	[− 10.5]	[− 12.2]

In general, the values of the chemshifts change monotonically with an increase in the oxidation state. Furthermore, the values support the observation that high oxidation states of Am and Cm were not reached in both clusters and the Pu$^{6+}$ state in YNbTiO$_6$ (the corresponding values are presented in parentheses).

### 3-center clusters for perfect crystals

Single-center clusters, while being easier to calculate, have inevitable limitations when a significant perturbation is introduced to the main cluster, such as the replacement of the center atom by an element with very different chemical properties and/or with different oxidation state/ionicity and atomic angular momenta for a given oxidation state. In such cases, an extended cluster model should rather be built. It can simulate not only relaxation in a larger area, but also additional defects such as charge-compensating vacancy.

**Figure 8:**
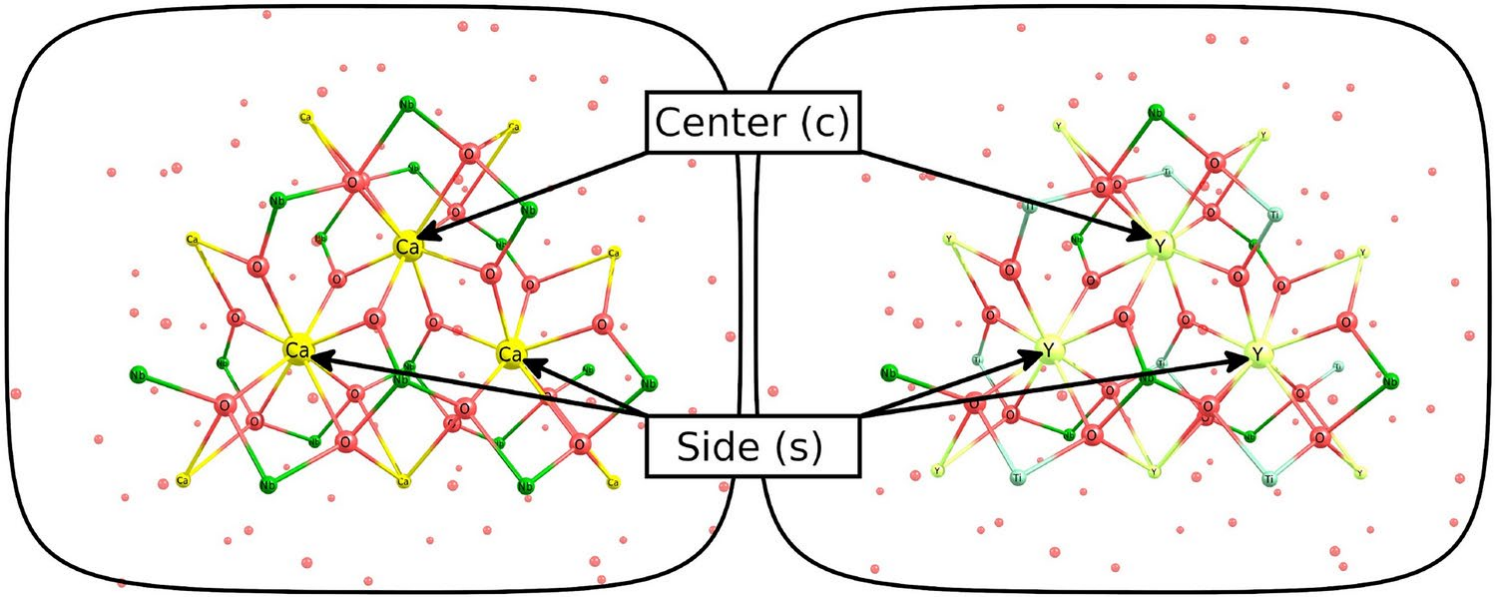
3-center clusters for Ca in CaNb$_2$O$_6$ (left) and Y in YNbTiO$_6$ (right). The “center” and “side” positions are denoted.

For a more detailed study of actinides in niobates, 3-center clusters were constructed for both crystal systems (Fig. 8). The clusters have the C$_2$ symmetry, which is, however, slightly distorted for YNbTiO$_6$ due to the decrease of the original crystal symmetry. For convenience, calcium centers are denoted as “center” and “side” below.

The remaining RMS forces on the main clusters after their geometry optimization were about two orders of magnitude larger than those for the single-center model. Although such values are still on par with common DFT errors, we have managed to improve the results several times by introducing and optimizing lc-CTPPs in NAE pseudoatoms (Table 4).

**Table 4:** Forces on the atoms of the main cluster.

Structure	RMS force (a.u.)
3c-Ca_3_ (CaNb_2_O_6_); no PP at NAE	5.7 · 10^-3^
3c-Ca_3_ (CaNb_2_O_6_); PP at NAE	1.1 · 10^-3^
3c-Y_3_ (YNbTiO_6_); no PP at NAE	5.8 · 10^-3^
3c-Y_3_ (YNbTiO_6_); PP at NAE	1.7 · 10^-3^

### Uranium in 3-center clusters

The 3-center clusters were used to simulate Ca$^{2+}$ $\rightarrow$U$^{n+}$ and Y$^{3+}$ $\rightarrow$U$^{n+}$ (n = 3,4,5,6) substitutions in CaNb$_2$O$_6$ and YNbTiO$_6$, respectively. Depending on the difference between the oxidation states of the substitute uranium and the original atom, several clusters were prepared. First, no vacancy clusters were built for all possible considered cases, neutral for Y$^{3+}$ $\rightarrow$U$^{3+}$ substitution, and positively charged for all other cases. As the 1-center clusters, the above-discussed clusters represent isolated crystal defects but allow more structural and electronic relaxation of their surroundings. Furthermore, if the substitution charge difference is greater than or equal to the oxidation state of the original atom (n$\ge$4 for CaNb$_2$O$_6$ and n = 6 for YNbTiO$_6$), clusters with a single vacancy were built using three arrangements with a substitute, original atom, or vacancy at the “center” position. For the Ca$^{2+}$ $\rightarrow$U$^{6+}$ substitution in CaNb$_2$O$_6$, a neutral cluster with two vacancies was also built.

**Figure 9:**
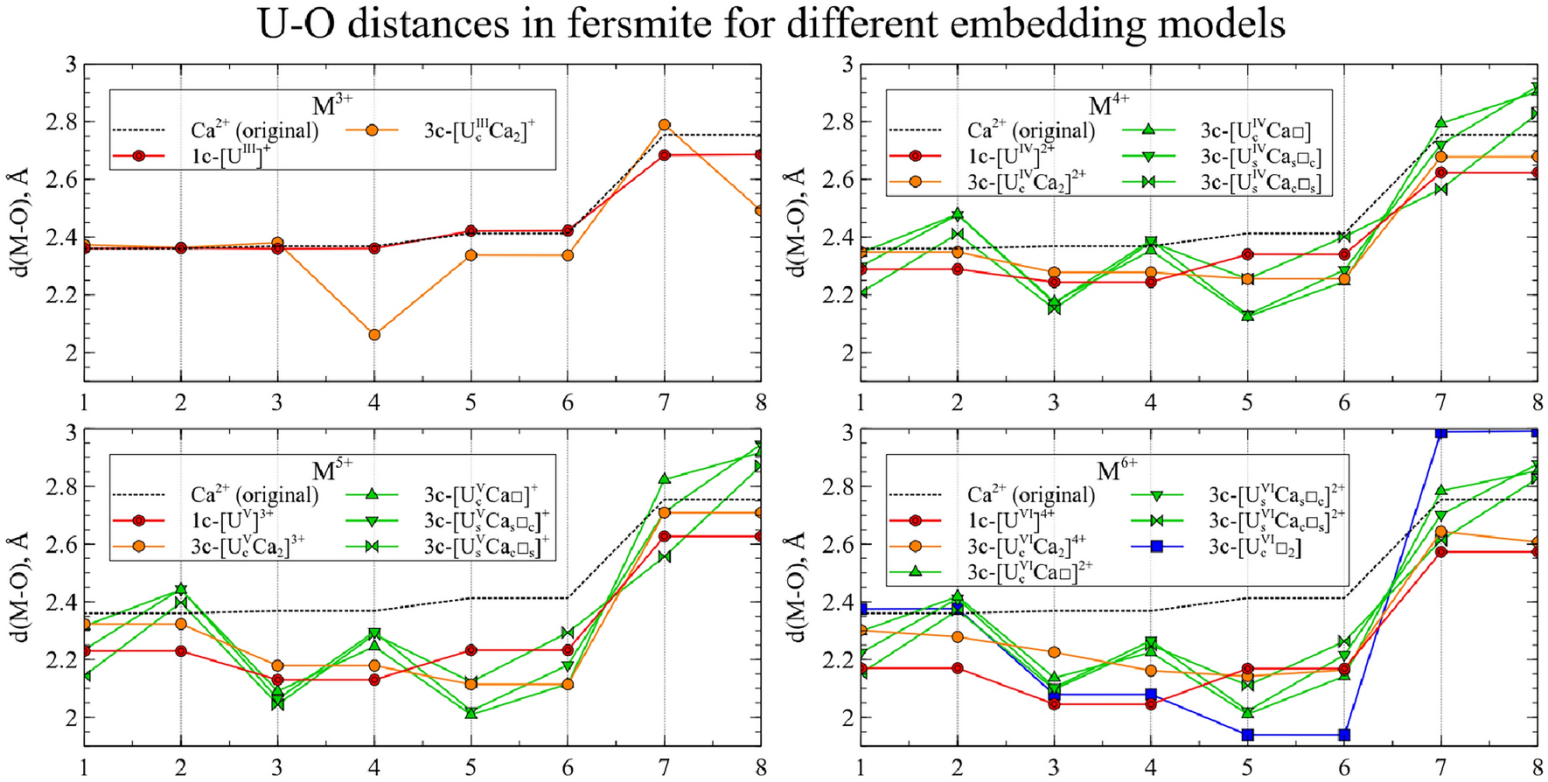
U-O distances for U substitutions in 3-center clusters for CaNb$_2$O$_6$. Numbers on x axis represent index of each O neighbor of the central atom. Dashed line corresponds to Ca-O distances in perfect crystals. Red lines (solid and dashed) represent U-O distances in a one-center cluster substitution model.

**Figure 10:**
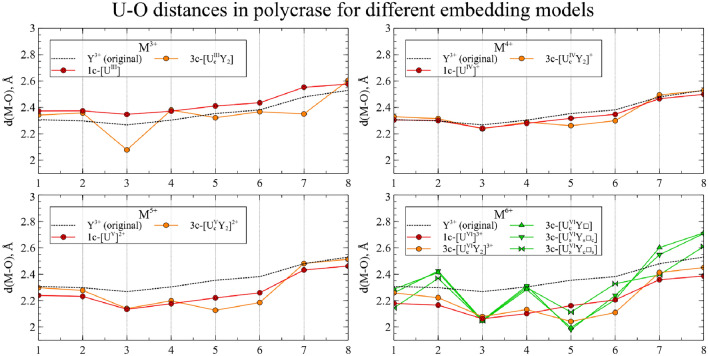
U-O distances for U substitutions in 3-center clusters for YNbTiO$_6$. Numbers on x axis represent index of each O neighbor of the central atom. Dashed line corresponds to Y-O distances in perfect crystals. Red lines (solid and dashed) represent U-O distances in a one-center cluster substitution model.

The U-O distances in the relaxed main clusters are presented in Figs. 9 and 10. For comparison, the distances for the perfect crystal and U substitutions in a single-center cluster model are also given.

Several conclusions can be drawn from the analysis of the resulting structures. For Ca$^{2+}$/Y$^{3+}$ $\rightarrow$U$^{4+}$ and Ca$^{2+}$/Y$^{3+}$ $\rightarrow$U$^{5+}$ substitutions, the minimal 1-center model (red line) and the charged 3-center model without vacancies (orange line) yield very similar structures, which means that the minimal model is sufficient here to simulate an isolated substitution. As expected, the neighboring vacancy distorts the structure; however, all 3 arrangements of the U atom and the vacancy (green lines) result in similar structures. In general, two cases of isolated substitution and the replacement compensated by a vacancy should be considered when studying such substitutions; whereas our multi-center models allow one to simulate both cases.

For the starting oxidation state with n = 3, the resulting structures without vacancies undergo significant distortion: several M-O distances become closer to that of the 1-center cluster with n = 3, several others become closer to that of the 1-center cluster with n = 4 and the remaining two distances are deviating from both of these clusters. For the starting oxidation state with n = 6, the cluster geometry without vacancies is closer to that of the 1-center model with n = 6 in the case of YNbTiO$_6$, but for CaNb$_2$O$_6$ the corresponding cluster is closer to the 1-center model with n = 5. The clusters with vacancies, as expected, undergo significant structural relaxation, but nevertheless one can notice that there is a considerable difference in geometry between the clusters with vacancies for n = 4 and n = 5, but the structures for n = 5 and n = 6 have only small differences. Both of these observations indicate that charge transfer takes place with oxidation and reduction of U$^{3+}$ and U$^{6+}$, respectively.

**Figure 11:**
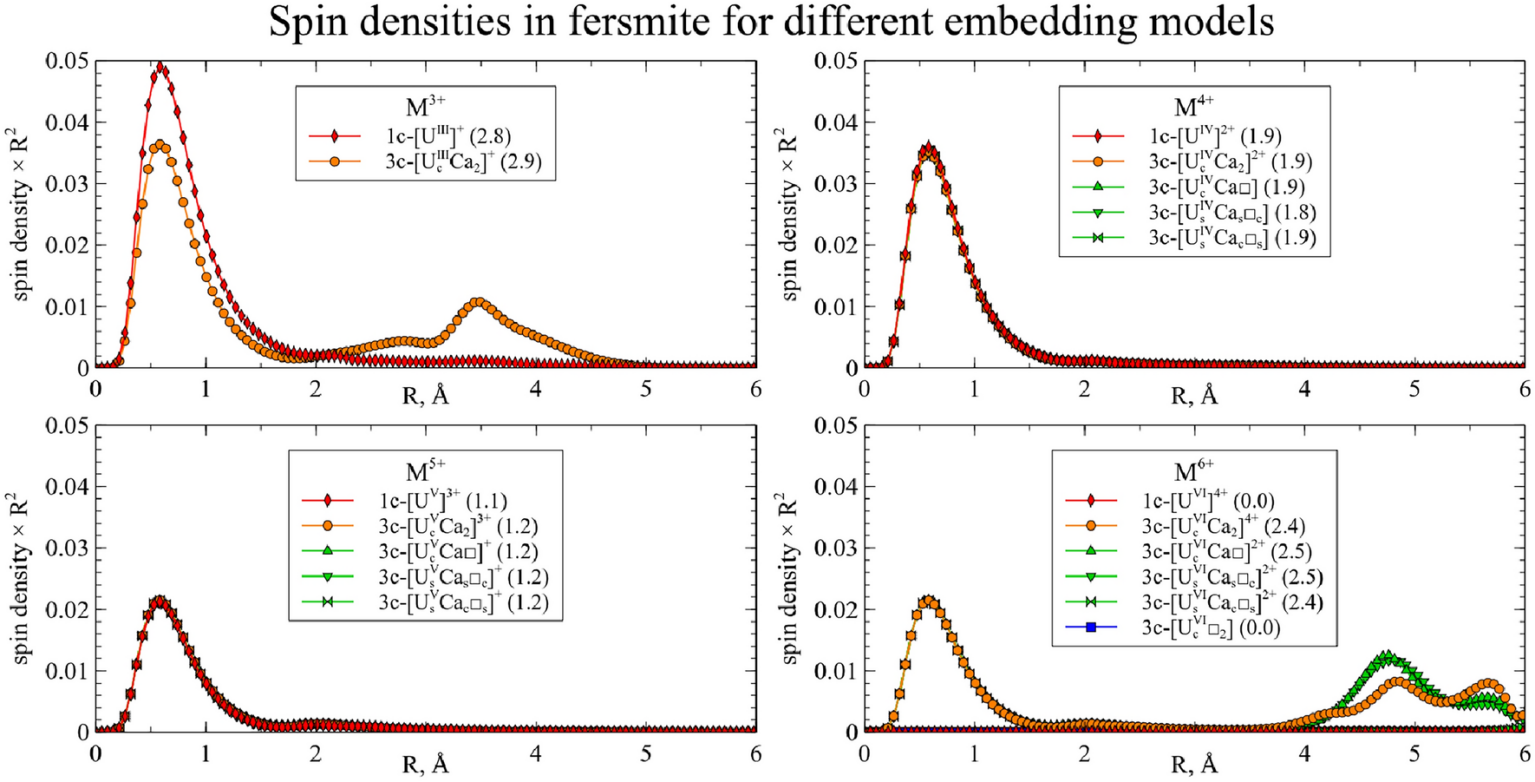
Integrated spin densities for U substitutions in 3-center clusters in CaNb$_2$O$_6$. Total values of integrated spin density are given in parentheses in the legend.

**Figure 12:**
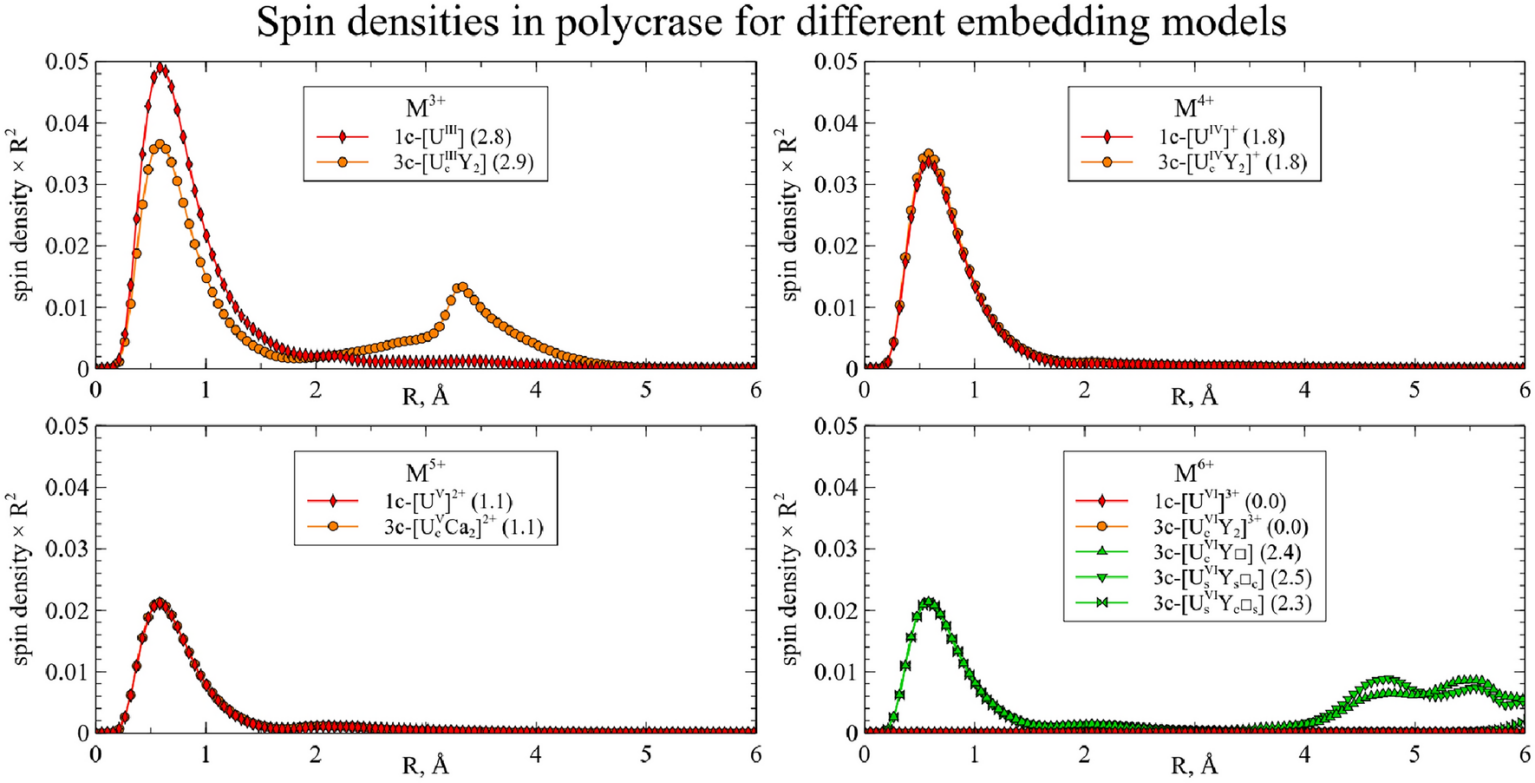
Integrated spin densities for U substitutions in 3-center clusters in YNbTiO$_6$. Total values of integrated spin density are given in parentheses in the legend.

Next, we performed an analysis of the spin densities, which were calculated and integrated for each cluster (Figs. 11 and 12).

As expected, for the U$^{4+}$ and U$^{5+}$ substitutions in both crystals, the spin densities obtained in the 3-center clusters match the spin densities for the corresponding 1-center clusters: the spin density is centered on the uranium atom and corresponds to a single unpaired electron for the U$^{5+}$ and two unpaired electrons for the U$^{4+}$ substitutions. For CaNb$_2$O$_6$, all the arrangements of clusters with a vacancy also yield the same spin density distribution.

For the U$^{3+}$ substitution in both clusters, the spin density on the uranium atom matches the spin density of the 1-center U$^{4+}$ cluster, and an additional peak appears on the distance about 3.5Å from the U atom, which implies that the U atom undergoes oxidation and its electron is transferred to the other atom (or group of atoms). From the integrated 1D graph it is hard to deduce which atoms or pseudoatoms are involved here, so we investigate 3D distributions below.

For the U$^{6+}$ case, the reverse electron transfer occurs: instead of the expected closed-shell U$^{6+}$ in most structures, the spin density distribution matches that of U$^{5+}$ with a single unpaired electron. Moreover, several spin density peaks arise at distances of 4.5÷6Å. Compared to the analogous results with 1-center clusters for Am, Cm, and Pu, it could be seen that the secondary peaks for U are considerably farther from the actinide atom. This can be explained by the assumption that the electron is transferred from the neighbor oxygen atoms for the 1-center clusters for Am, Cm, and Pu, and from the non-neighbor atoms for the 3-center clusters for U. Once again, the 3D distribution is required to study these electron transfers in detail. There are, however, two exceptions: the cluster without vacancies for YNbTiO$_6$ and the cluster with two vacancies for CaNb$_2$O$_6$ result in a zero spin density, corresponding to the U$^{6+}$ state.

In general, the comparison of structures and electronic densities can be interpreted in such a way that for a simple Y/Ca$\rightarrow$U$^{n+}$ substitution only the structures with n = 4,5 are viable, while U$^{3+}$ and U$^{6+}$ undergo oxidation and reduction, respectively. The latter seems to contradict the fact that U$^{\textrm{VI}}$ was indeed found in niobate matrices^5^, however, it can be explained by the assumption that U$^{\textrm{VI}}$ in niobates does not exist as a simple substitution, but in the form of uranyl cation in a metamict phase.

**Figure 13:**
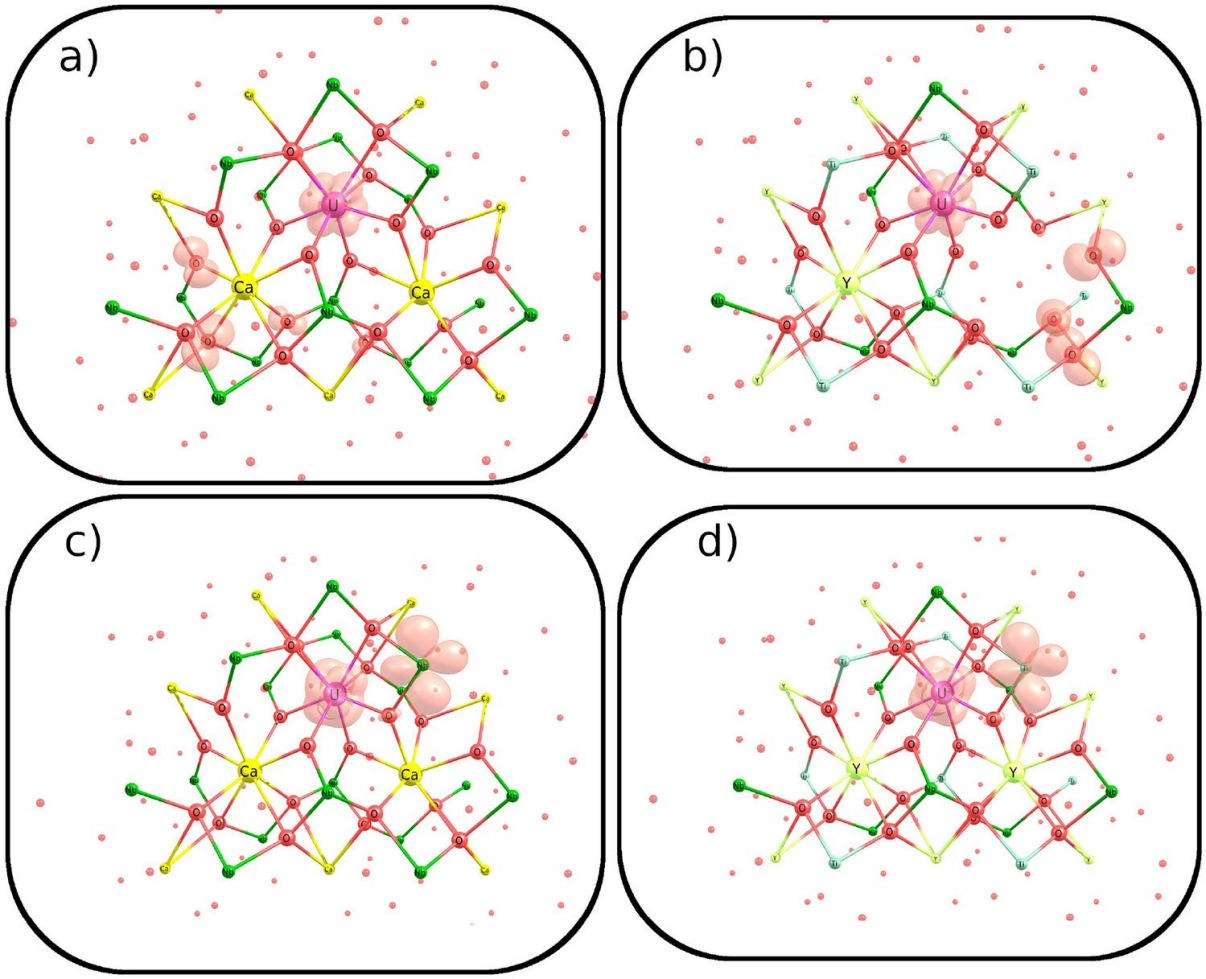
Three-dimensional pictures of spin densities for 3-center clusters with uranium substitutes: (**a**) Ca$^{2+}$ $\rightarrow$U$^{6+}$ in CaNb$_2$O$_6$; (**b**) 2Y$^{3+}$ $\rightarrow$U$^{6+}$+vacancy in YNbTiO$_6$; (**c**) Ca$^{2+}$ $\rightarrow$U$^{3+}$ in CaNb$_2$O$_6$; (**d**) Y$^{3+}$ $\rightarrow$U$^{3+}$ in YNbTiO$_6$.

For a more detailed visualization of the nature of the electronic transfers mentioned above, 3D spin density plots are presented in Fig. 13.

**Figure 14:**
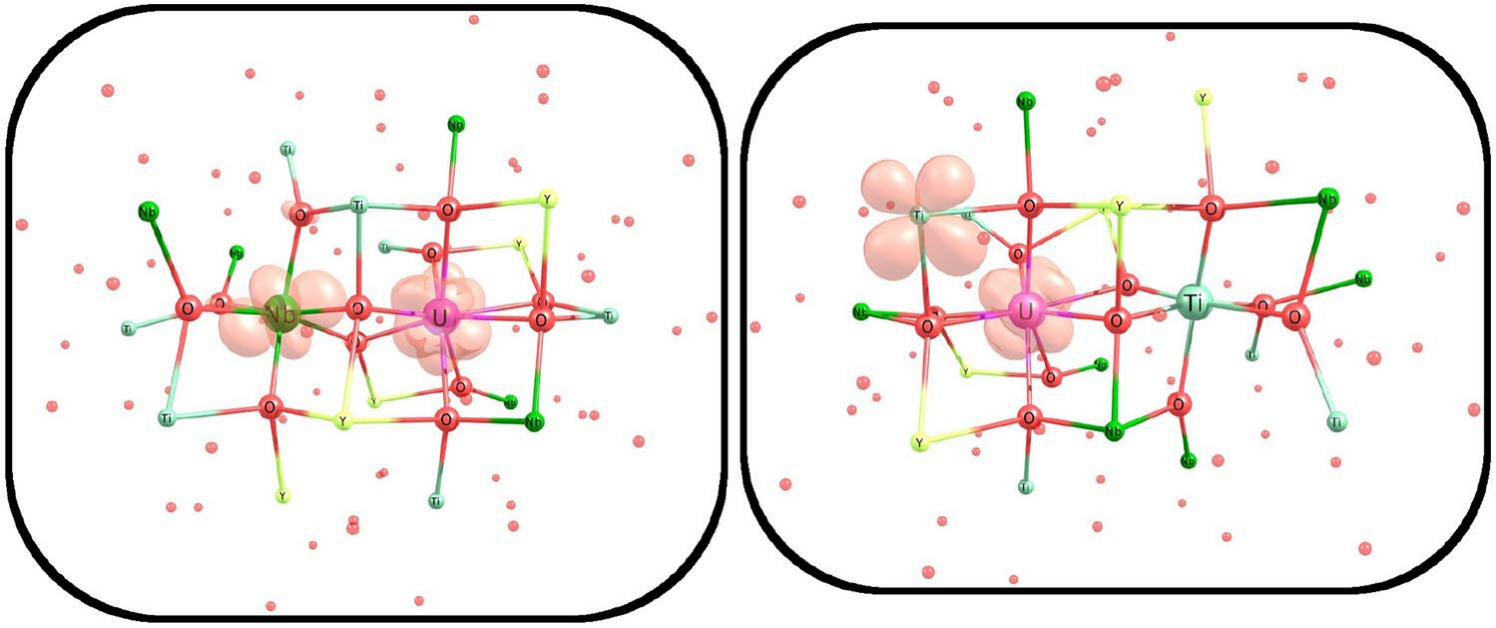
Spin densities for Y$^{3+}$ $\rightarrow$U$^{3+}$ substitution in YNbTiO$_6$ for clusters with Y,Nb and Y,Ti centers.

For the U$^{6+}$ substitution in both clusters, the O$\rightarrow$U electron transfer takes place; however, this does not come from directly adjacent oxygen atoms, but from neighbors of another center or vacancy. This can be explained in terms of the lower oxidation potential of U$^{6+}$ compared to Am$^{6+}$, Cm$^{6+}$, and Pu$^{6+}$ ions, for which electron transfers occur even in 1-center clusters of the direct neighbor oxygen atoms. For U$^{3+}$, there is an electron transfer in the opposite direction: from U to the pseudoatom of the cationic layer in the CTEP model. The latter raises the question: is such electron transfer to the pseudoatom an indication of possible transfer to a real atom in the original crystal or is it an error of the CTEP model? To estimate the ability of electron transfer to real Ti and Nb atoms (that is, to those located inside the main cluster), two 2-center embedded clusters were built: with Y,Nb, and Y,Ti centers. In each cluster Y$^{3+}$ $\rightarrow$U$^{3+}$ were simulated with a subsequent relaxation of the structure. The corresponding 3D spin density plots are presented in Fig. 14.

For the U, Ti cluster, the U$\rightarrow$Ti electron transfer was not observed, the same transfer to the pseudoatom was found instead as in the 3-center clusters. However, for the U,Nb cluster, U$\rightarrow$Nb transfer occurred. The latter allows us to conclude that U$^{3+}$ is a strong electron donor which cannot exist in niobate matrices without being oxidized. Although the Ti pseudoatom (simulated by lc-CTPP and the partial point “pseudo-nuclear charge”) was indeed found to be a stronger electron acceptor than a realistic Ti atom model (treated by *ab initio* small core PP and the corresponding basis set), the same Ti pseudoatom was found to be a weaker acceptor than a realistic Nb atom model, so the difference is not significant enough to invalidate the CTEP model.

The other question concerns the electronic transfer paths between the vacancy and the uranium atoms. However, a reliable answer to the question requires a more sophisticated study of possible paths involving the wave function theory (Fock-space coupled cluster methods^92,93^ as is used in^72^ with pilot application to Th and Ce impurities in xenotime; or more economical but potentially applicable for actinides versions of perturbation theory, for example, multipartitioning methods^94,95^) in the vicinity of the pass point. Both of these approaches are being developed to increase their efficiency, and their use is planned elsewhere in the near future. In turn, information on equilibrium structures with impurity actinides is of interest in the work mainly for the problem of long-term immobilization of radioactive waste; therefore, the study of electron transfer processes in this particular case is not so important because the local structure of the impurity center with vacancies is probably formed quite soon after the stage of incorporation of actinides into the crystal.

### Nb and Ti clusters

As mentioned above, while we use perfect crystals for our calculations, the Nb and Ti atoms in the practically synthesized YNbTiO$_6$ structure are randomly arranged. The embedding potential method allows one to estimate the short-range effects of such a rearrangement by modeling it as one or more substitutions in a corresponding cluster. In the given section, we make a comparison of our CTEP model of polycrase with the SQS one. In the present study, three clusters were built: minimal single-center clusters NbO$_6$@CTEP, TiO$_6$@CTEP, and double-center NbTiO$_{10}$@CTEP cluster; five substitutions in total were modeled with consequent relaxation of geometry:

1. Nb@CTEP $\rightarrow$ Ti@CTEP$^-$
2. Ti@CTEP $\rightarrow$ Nb@CTEP$^+$
3. NbTi@CTEP $\rightarrow$ Nb$_2$@CTEP$^+$
4. NbTi@CTEP $\rightarrow$ Ti$_2$@CTEP$^-$
5. NbTi@CTEP $\rightarrow$ TiNb@CTEP (swap)

**Figure 15:**
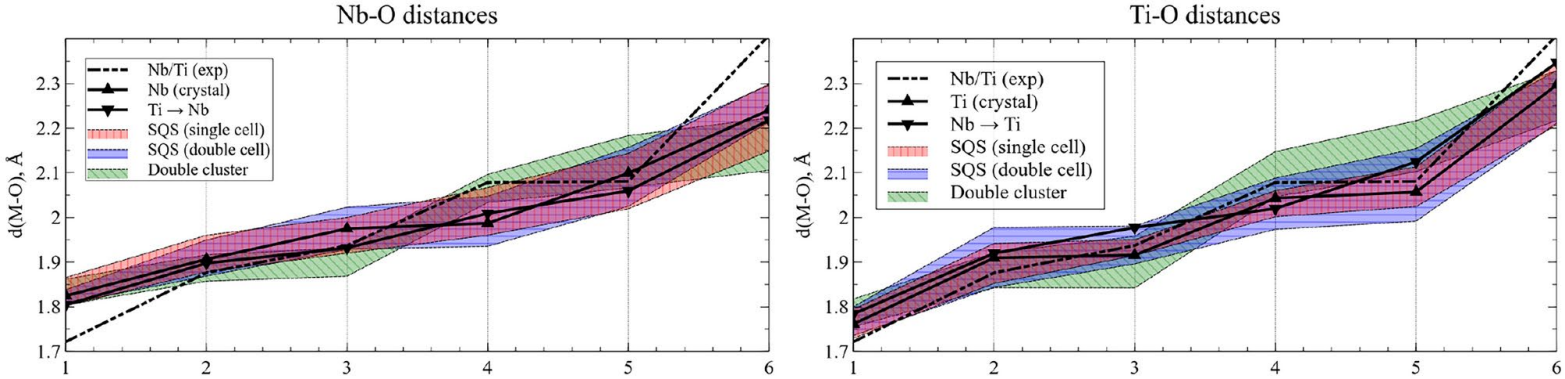
Structures of Nb/Ti substitutions in YNbTiO$_6$ compared with experimental and SQS structures. SQS and double cluster (NbTi) substitutions structures are presented as ranges.

The SQS method was applied to a single cell and a double supercell with the use of the package sqsgenerator^96^. For both resulting structures, relaxation of geometry was performed with the same parameters as for the high-symmetry cell chosen to build CTEP clusters. The results are displayed in Fig. 15.

As follows from the graph, the local structures of the SQS model exhibit the variation of each individual Ti / Nb-O distance within the range of $0.1\div0.15$ Å. For both Nb and Ti, the experimental distance to the farthest O atom lies far outside the SQS range, and for Nb, this is the same for the closest O atom, which indicates that an averaged experimental structure is only a rough approximation to the real one. The SQS variations for single and double cells are close to each other; however, the single-cell variation is slightly larger for the Nb-O distances and smaller for the Ti-O distances. The distances for the crystal calculation of the high-symmetry cell and for both Ti$\rightarrow$ Nb and Nb$\rightarrow$ Ti lie within the SQS range; however, the distance range of double-cluster substitutions is considerably different from the SQS one. The reason for the latter discrepancy is unclear yet and requires further investigation. Overall, while it is apparent that the local environment of the atom in a disordered structure cannot be modeled by a single cluster, the chosen crystal cell and the CTEP clusters are capable of providing fairly representative data with a reasonable computational effort.

## Conclusions

The CTEP method is applied to study actinide substitutions in two niobate crystals, CaNb$_2$O$_6$ and YNbTiO$_6$. Two one-center clusters are built (with Ca and Y centers, respectively), and 20 substitutions (five actinides, each in four different oxidation states) were made for each cluster. Geometry relaxation is performed for each resulting structure, and electronic properties are analyzed by evaluating the spin density distribution and calculating the chemical shifts of $K_{\alpha }$ lines of X-ray emission spectra. In general, the studied embedded clusters with actinides having the same oxidation state are found to yield similar local structure distortions that indicate the similarity of the behavior of these elements in the studied niobate matrices.

However, in some cases, the electron transfer was found to take place. Am and Cm in high oxidation “starting” states accept electrons from neighboring O atoms, while “starting” U$^\textrm{III}$ donates an electron to the second-order (cationic) neighbors. Both results indicate that the mentioned elements cannot exist in these oxidation states in the studied niobate matrices and undergo reduction or oxidation, respectively.

The U substitutions are additionally studied with the use of multicenter models, which can provide both more structural and electronic relaxation and also include charge-compensating vacancies. For “starting” U$^\textrm{III}$ state, the electron transfer to the Nb cation is confirmed. For “starting” U$^\textrm{VI}$ state, the reduction similar to that of Am$^\textrm{VI}$ and Cm$^\textrm{VI}$ in the one-center clusters was found, except that the electron is donated by oxygen atoms from the third-order coordination sphere and not by the nearest ones. This result seems to contradict the experimental data since U$^\textrm{VI}$ was found in niobates; however, the contradiction can be explained by the fact that U$^\textrm{VI}$ in practice exists not in a perfect crystal phase, but in the metamict one.

Furthermore, since the practically synthesized YNbTiO$_6$ structures cannot be considered perfect (periodic) crystals because the Nb and Ti atoms are statistically distributed within them occupying the same Wyckoff positions, different Ti $\leftrightarrow$ Nb substitutions are studied and the corresponding structural changes are estimated.

Although all calculations in the present study are performed within the DFT framework, the CTEP method, being applied to crystal fragments of moderate sizes, allows one to use more sophisticated wave-function based methods, which we are going to apply to the niobate crystals in our future studies similar to that in xenotime^72^.

## Supplementary Information

Supplementary Information.

## Data Availability

Structures and partial charges of the single-center CTEP clusters and the corresponding pseudopotentials and basis sets are presented in the supplementary file. Pseudopotentials and basis sets for actinide atoms are available at http://qchem.pnpi.spb.ru/recp. Rest of the data is available from the corresponding author on reasonable request.
