# Supplementary material for: Electronic structure study of YNbTiO[image] and CaNb[image]O[image] with actinide impurities using compound-tunable embedding potential method
Source: Sci Rep. 2025 Mar 27;15:10645. doi: 10.1038/s41598-025-94297-3 (PMC11950246; doi:10.1038/s41598-025-94297-3)
Supplement: Supplementary file 1 — Supplementary Information. [file 41598_2025_94297_MOESM1_ESM.pdf]

Supplementary materials for article: Electronic structure study of $YNbTiO_6$ vs. $CaNb_2O_6$ with U, Pu and minor actinide substitutions using compound-tunable embedding potential method

D.A. Maltsev[1,*], Yu.V. Lomachuk[1], V.M. Shakhova[1], N.S. Mosyagin[1], D.O. Kozina[1], and A.V. Titov[1,+]

[1]Petersburg Nuclear Physics Institute named by B.P. Konstantinov of National Research Center “Kurchatov Institute” (NRC “Kurchatov Institute” - PNPI), 188300, Russian Federation, Leningrad district, Gatchina, mkr. Orlova roscha, 1.
[*]malcev_da@pnpi.nrcki.ru
[+]titov_av@pnpi.nrcki.ru

Table S1. Pseudopotentials used for all atoms in solid-state calculations and main cluster atoms in CTEP calculations

	Ca				Nb				Ti				Y		
	n_{core} = 10; l_{max} = 2				n_{core} = 28; l_{max} = 3				n_{core} = 10; l_{max} = 2				n_{core} = 28; l_{max} = 3		
	Exponent	r^n	Coefficient		Exponent	r^n	Coefficient		Exponent	r^n	Coefficient		Exponent	r^n	Coefficient
d	1662.91620	1	-1.03094	f	53.95222	1	-9.29245	d	22.62976	1	-8.34325	f	11.81594	1	-12.91092
	193.50801	1	-2.83669		39.96834	1	29.77031		33.03067	2	24.35487		5.59889	2	-11.18023
	42.49352	1	-2.24294		29.73805	1	-24.13418		10.44300	2	-3.08608		2.06958	2	-0.85745
	13.18275	1	-3.22888		7.99197	1	-7.95015		5.33823	2	-0.66926		1.43176	2	0.14793
	0.88412	1	-1.16370		3.05366	1	-1.32608		0.84402	2	0.01365		0.97068	2	-0.22925
	2.14318	2	1.12110		0.94465	2	-0.00993		0.28954	2	0.00261				
	0.99739	2	0.93384		0.58492	2	0.00266								
	0.24066	2	0.00231												
s-d	49.10184	0	3.00000	s-f	0.54109	0	6.00000	s-d	0.54335	0	3.00000	s-f	20.03229	0	6.00000
	9.82328	1	102.25002		7.59264	1	125.76704		8.55278	1	-32.02722		6.42649	1	194.36171
	7.11472	1	-200.43483		5.42717	1	-325.20472		0.59983	1	64.80225		4.68992	1	-350.17035
	5.16341	1	172.00211		3.80989	1	394.03796		2.84126	2	-78.36560		3.37948	1	330.47086
	3.69557	1	-41.86718		2.59190	1	-258.27611		1.18063	2	-44.76135		1.17384	1	-159.06590
	0.61002	1	2.15841		1.79224	1	131.95900		0.66455	2	-36.27391		1.39457	2	163.34693
	0.76875	2	-1.92302		1.26164	1	-56.14178		0.47915	2	-0.58841		0.66959	2	2.22323
	0.38155	2	-0.04288		0.89264	1	14.01572						0.44327	2	-0.34080
					0.62920	1	-5.90487						0.27351	2	0.16679
p-d	0.90417	0	2.00000	p-f	0.86511	0	5.00000	p-d	0.97328	0	2.00000	p-f	6.35136	0	5.00000
	6.28202	1	-13.46518		7.26898	1	92.37831		9.32294	1	19.63807		9.25965	1	16.35956
	0.23791	1	29.75614		5.22894	1	-214.30259		6.82564	1	-94.87431		2.40646	2	87.70585
	2.03486	2	-29.57636		3.83044	1	206.29052		5.02891	1	91.11808		1.74971	2	-93.62825
	0.82940	2	-15.39273		2.79670	1	-63.00349		4.09271	2	-52.11260		1.26880	2	54.35196
	0.40034	2	-10.22392		0.50063	1	-3.87193		0.97328	2	-1.60534		0.91212	2	-20.44031
	0.25441	2	-8.64993		0.55104	2	1.99654		0.66936	2	0.53616		0.64863	2	5.08534
	0.17872	2	-0.01330						0.45536	2	-0.11373		0.45450	2	-0.77713
									0.30200	2	0.00670		0.31152	2	0.05389
				d-f	0.79689	0	3.00000					d-f	0.58493	0	3.00000
					13.37612	1	13.21495						10.59573	1	9.82548
					1.70959	1	2.62233						4.44226	1	4.52887
					6.35170	2	11.83490						1.44402	1	1.46088
					1.18477	2	-3.60134						0.85507	2	-2.36137

Table S2. Basis sets used for all atoms in solid-state calculations and main cluster atoms in CTEP calculations

	Ca (5,4,1)/[4,4,1]		Nb (5,5,5,1)/[4,4,3,1]		Ti (5,4,5,1)/[5,4,5,1]		Y (5,5,4,1)/[5,5,4,1]		O (10,6,1)/[4,3,1]	
	Exp	Coeff	Exp	Coeff	Exp	Coeff	Exp	Coeff	Exp	Coeff
s	1.39705	2.21138	5.69882	-0.35440	3.35929	1.00000	4.09958	1.00000	27032.38263	0.00057
	1.11767	-3.14468	3.34266	1.32989	2.58407	1.00000	3.40192	1.00000	4052.38714	0.00444
	0.68395	1.00000	1.78998	1.00000	0.91790	1.00000	1.29828	1.00000	922.32723	0.02302
	0.34448	1.00000	0.77871	1.00000	0.48080	1.00000	0.82211	1.00000	261.24071	0.09282
	0.14015	1.00000	0.35874	1.00000	0.18000	1.00000	0.34991	1.00000	85.35464	0.29379
									31.03504	0.67402
									12.26086	0.63840
									4.99871	0.39535
									1.09871	1.00000
									0.35659	1.00000
p	2.88020	1.00000	14.40461	0.01330	6.26809	1.00000	3.00000	1.00000	63.27495	0.01202
	0.73397	1.00000	2.99750	0.99334	2.25766	1.00000	1.85000	1.00000	14.62705	0.08301
	0.31966	1.00000	1.96576	1.00000	0.96823	1.00000	0.91760	1.00000	4.45012	0.31992
	0.15030	1.00000	0.80836	1.00000	0.37057	1.00000	0.44067	1.00000	1.52758	0.70716
			0.30000	1.00000			0.19193	1.00000	0.54897	1.00000
									0.18587	1.00000
d	0.98864	1.00000	30.21561	-0.00387	10.63409	1.00000	2.35071	1.00000	0.25346	1.00000
			8.67166	-0.03331	3.65443	1.00000	1.95079	1.00000		
			1.66428	1.01101	1.35886	1.00000	0.61300	1.00000		
			0.78654	1.00000	0.86368	1.00000	0.18198	1.00000		
			0.34988	1.00000	0.33184	1.00000				
f			0.52270	1.00000	0.56200	1.00000	0.65147	1.00000		

Table S3. Pseudopotentials used for NCE pseudoatoms for CTEP calculations for $YNbTiO_6$. For convenience, original elements are replaced by pseudoatoms

	Kr (Ti_{CTEP}) n_{core} = 36; l_{max} = 2				Ne (Nb_{CTEP}) n_{core} = 10; l_{max} = 4				Ar (Y_{CTEP}) n_{core} = 18; l_{max} = 3		
	Exponent	r^n	Coefficient		Exponent	r^n	Coefficient		Exponent	r^n	Coefficient
d	3.58018	1	31.54390	g	45.04716	1	-0.39166	f	38.79776	1	-14.03979
	1.20182	1	-50.65838		2.88945	1	-5.37483		4.45365	1	-15.34666
	1.52688	2	60.42780		1.89725	1	9.97223		0.98283	2	-5.93978
					1.03930	1	-9.58213				
					0.55036	1	3.25426				
s-d	2.96180	0	3.00000	s-g	2.05770	0	6.00000	s-f	3.63835	0	6.00000
	25.10863	1	-0.81958		8.08518	1	-1.90334		1.22681	1	30.62957
	0.70455	1	1.36978		2.09266	1	-3.44114		0.41090	1	-30.35815
	0.80723	2	6.77011		0.57556	1	13.27274		0.52089	2	27.63167
	3.58018	1	-31.54390		0.33332	1	-2.43637		38.79776	1	14.03979
	1.20182	1	50.65838						4.45365	1	15.34666
	1.52688	2	-60.42780						0.98283	2	5.93978
p-d	7.05634	0	2.00000	p-g	0.61934	0	5.00000	p-f	1.68975	0	5.00000
	1.99441	1	17.56715		0.45463	1	-0.90247		4.41077	1	-3.75395
	0.62920	1	-17.79313		0.31118	1	10.03578		0.28283	1	5.73122
	0.69963	2	19.64764		0.22255	1	-5.75506		0.17739	2	-0.25154
	3.58018	1	-31.54390		0.15799	1	1.09857		38.79776	1	14.03979
	1.20182	1	50.65838						4.45365	1	15.34666
	1.52688	2	-60.42780						0.98283	2	5.93978
				d-g	3.09053	0	3.00000	d-f	2.48918	0	3.00000
					8.20687	1	-3.64768		7.97147	1	-2.62931
					5.86174	1	2.91814		0.52689	1	-2.42147
					1.17397	1	1.01157		0.17747	2	-0.05439
					0.35374	1	-2.10483		38.79776	1	14.03979
									4.45365	1	15.34666
									0.98283	2	5.93978
				f-g	10.82138	1	-11.45942				
					2.03469	1	-9.04666				
					0.75726	1	4.55203				
					0.52390	1	-3.03324				
					0.34253	1	0.03535				

Table S4. Basis sets used for NCE pseudoatoms for CTEP calculations for $YNbTiO_6$. For convenience, original elements are replaced by pseudoatoms

	Kr (Ti_{CTEP}) (4,2,5,1)/[4,2,5,1]		Ne (Nb_{CTEP}) (3,2,4,1)/[3,2,3,1]		Ar (Y_{CTEP}) (3,2,4,1)/[3,2,3,1]	
	Exp	Coeff	Exp	Coeff	Exp	Coeff
s	2.30594	1.00000	2.04788	1.00000	1.44076	1.00000
	1.00176	1.00000	0.91802	1.00000	0.82211	1.00000
	0.48080	1.00000	0.35874	1.00000	0.34991	1.00000
	0.18000	1.00000				
p	0.87167	1.00000	0.78954	1.00000	0.44067	1.00000
	0.37057	1.00000	0.30000	1.00000	0.19193	1.00000
d	10.63409	1.00000	4.37984	-0.13153	3.28664	-0.07807
	3.65443	1.00000	1.90403	1.09404	1.44255	1.05716
	1.35886	1.00000	0.82773	1.00000	0.61300	1.00000
	0.86368	1.00000	0.34988	1.00000	0.18198	1.00000
	0.33184	1.00000				
f	0.56200	1.00000	0.52270	1.00000	0.65147	1.00000

Table S5. Pseudopotentials used for NCE pseudoatoms for CTEP calculations for $CaNb_2O_6$. For convenience, original elements are replaced by pseudoatoms

	Mg (Ca_{CTEP}) n_{core} = 12; l_{max} = 2				Ne (Nb_{CTEP}) n_{core} = 10; l_{max} = 4		
	Exponent	r^n	Coefficient		Exponent	r^n	Coefficient
d	75.97807	1	-3.49662	g	43.49700	1	-0.46187
	13.68134	1	-5.23166		3.07508	1	-4.89447
	2.66466	1	-3.29878		1.92300	1	8.57323
	0.78619	1	-2.97046		1.00627	1	-9.03867
	0.20587	1	-0.52534		0.54213	1	3.72314
s-d	16.50562	0	3.00000	s-g	2.12432	0	6.00000
	7.07837	1	8.89934		8.12754	1	-1.87284
	2.08699	1	8.89338		2.00705	1	-3.26760
	0.42226	1	7.86313		0.56659	1	14.78758
	0.15981	1	-0.18564		0.36630	1	-3.94198
p-d	19.54933	0	2.00000	p-g	1.01064	0	5.00000
	185.24700	1	15.95468		2.05422	1	-1.25262
	12.45052	1	14.14524		0.37940	1	7.37623
	2.89399	1	7.72415		0.19180	1	-1.87042
	0.95752	1	6.97232		0.13767	1	0.56524
	0.17066	1	3.74232				
				d-g	3.55275	0	3.00000
					231.49020	1	-0.18421
					12.44056	1	-1.53750
					2.00475	1	2.12489
					0.35534	1	-2.35889
				f-g	10.01098	1	-14.66872
					7.21362	1	3.93043
					2.12918	1	-9.39240
					0.72776	1	4.28234
					0.51272	1	-3.13363

Table S6. Basis sets used for NCE pseudoatoms for CTEP calculations for $CaNb_2O_6$. For convenience, original elements are replaced by pseudoatoms

	Mg (Ca_{CTEP}) (3,3,1)/[3,3,1]		Ne (Nb_{CTEP}) (3,2,4,1)/[3,2,3,1]	
	Exp	Coeff	Exp	Coeff
s	0.72000	1.00000	2.04788	1.00000
	0.34448	1.00000	0.91802	1.00000
	0.14015	1.00000	0.35874	1.00000
p	0.55000	1.00000	0.78954	1.00000
	0.31966	1.00000	0.30000	1.00000
	0.15030	1.00000		
d	0.98864	1.00000	4.37984	-0.13153
			1.90403	1.09404
			0.82773	1.00000
			0.34988	1.00000
f			0.52270	1.00000

Table S7. Expanded basis sets, used for testing CTEP transferability; real atoms

	Ca (6,6,5,1)/[5,6,5,1]		Y (8,7,6,2)/[8,7,6,2]		O (12,7,2)/[6,4,2]	
	Exp	Coeff	Exp	Coeff	Exp	Coeff
s	1.39705	2.21138	4.09958	1.00000	27032.38263	0.00057
	1.11767	-3.14468	3.40192	1.00000	4052.38714	0.00444
	0.68395	1.00000	1.29828	1.00000	922.32723	0.02302
	0.34448	1.00000	0.82211	1.00000	261.24071	0.09282
	0.14015	1.00000	0.34991	1.00000	85.35464	0.29379
	0.04273	1.00000	0.06655	1.00000	31.03504	0.67402
			0.02866	1.00000	12.26086	0.63840
			0.01000	1.00000	4.99871	0.39535
					1.17031	1.00000
					0.46475	1.00000
					0.18505	1.00000
					0.07896	1.00000
p	2.88020	1.00000	3.00000	1.00000	63.27495	0.01202
	0.73397	1.00000	1.85000	1.00000	14.62705	0.08301
	0.31966	1.00000	0.91760	1.00000	4.45012	0.31992
	0.15030	1.00000	0.44067	1.00000	1.52758	0.70716
	0.08488	1.00000	0.18382	1.00000	0.52935	1.00000
	0.02052	1.00000	0.06057	1.00000	0.17478	1.00000
			0.02224	1.00000	0.06856	1.00000
d	1.19611	1.00000	2.35071	1.00000	1.18500	1.00000
	0.92823	1.00000	1.95079	1.00000	0.33200	1.00000
	0.32163	1.00000	0.61300	1.00000		
	0.14820	1.00000	0.20018	1.00000		
	0.05951	1.00000	0.06119	1.00000		
			0.02000	1.00000		
f	0.53827	1.00000	0.20018	1.00000		
			0.02000	1.00000		

Table S8. Expanded basis sets, used for testing CTEP transferability; pseudoatoms

	Mg (Ca_{CTEP}) (4,5,4,1)/[4,5,4,1] Exp	Coeff	Ne (Nb_{CTEP}) (6,5,6,2)/[6,5,5,2] Exp	Coeff	Kr (Ti_{CTEP}) (5,3,5,1)/[5,3,5,1] Exp	Coeff	Ar (Y_{CTEP}) (6,4,6,2)/[6,4,5,2] Exp	Coeff
s	0.72000	1.00000	2.04788	1.00000	2.30594	1.00000	1.44076	1.00000
	0.34448	1.00000	0.91802	1.00000	1.00176	1.00000	0.82211	1.00000
	0.14015	1.00000	0.35874	1.00000	0.48080	1.00000	0.34991	1.00000
	0.04273	1.00000	0.13557	1.00000	0.08516	1.00000	0.06655	1.00000
			0.06276	1.00000	0.03266	1.00000	0.02866	1.00000
			0.02734	1.00000			0.01000	1.00000
p	0.55000	1.00000	0.78954	1.00000	0.87167	1.00000	0.44067	1.00000
	0.31966	1.00000	0.30000	1.00000	0.37057	1.00000	0.18382	1.00000
	0.15030	1.00000	0.15873	1.00000	0.10156	1.00000	0.06057	1.00000
	0.08488	1.00000	0.08253	1.00000			0.02224	1.00000
	0.02052	1.00000	0.03518	1.00000				
d	1.19611	1.00000	4.37984	-0.13153	10.63409	1.00000	3.28664	-0.07807
	0.92823	1.00000	1.90403	1.09404	3.65443	1.00000	1.44255	1.05716
	0.32163	1.00000	0.82773	1.00000	1.35886	1.00000	0.61300	1.00000
	0.09386	1.00000	0.34988	1.00000	0.49213	1.00000	0.20018	1.00000
			0.14820	1.00000	0.16331	1.00000	0.06119	1.00000
			0.05951	1.00000			0.02000	1.00000
f	0.53827	1.00000	0.88801	1.00000	0.56200	1.00000	0.20018	1.00000
			0.31900	1.00000			0.02000	1.00000

Table S9. Cluster structures and partial charges for CaO_8@CTEP and YO_8@CTEP. For convenience, original elements are replaced by pseudoatoms (see previous tables)

		CaO_8@CTEP					YO_8@CTEP		
Atom type	x, Å	y, Å	z, Å	partial charge	Atom type	x, Å	y, Å	z, Å	partial charge
Ca	0.00000	1.28747	-1.30261	–	Y	1.33275	-0.00453	1.31279	–
O	1.33773	0.55800	-3.11622	–	O	-0.16702	-1.35633	2.41006	–
O	-1.49015	2.49607	-2.67747	–	O	-0.42081	1.25002	0.50070	–
O	-1.33773	0.55800	0.51099	–	O	0.42081	-1.25002	-0.50070	–
O	-1.33773	-0.55800	-2.09424	–	O	2.14674	1.27629	-0.48658	–
O	1.33773	-0.55800	-0.51099	–	O	2.42303	-1.35960	3.08018	–
O	1.49015	2.49607	0.07225	–	O	0.22060	1.35633	3.13254	–
O	-1.49015	3.25108	-0.07225	–	O	2.79616	1.35960	2.46242	–
O	1.49015	3.25108	-2.53298	–	O	3.01887	-1.27629	0.48658	–
Ne (Nb$_{\mathrm{CTEP}}$)	2.48697	1.81041	-3.63786	4.27600	Kr (Ti$_{\mathrm{CTEP}}$)	-1.61066	-2.41149	1.72233	3.53200
Mg (Ca$_{\mathrm{CTEP}}$)	0.00000	-1.28747	-3.90784	1.17326	Ne (Nb$_{\mathrm{CTEP}}$)	0.95586	-2.47968	3.78289	4.15978
Ne (Nb$_{\mathrm{CTEP}}$)	-2.48697	1.81041	-4.17782	3.94399	Ar (Y$_{\mathrm{CTEP}}$)	-1.27917	0.00453	4.22981	2.50082
Mg (Ca$_{\mathrm{CTEP}}$)	0.00000	4.45969	-3.90784	1.72171	Ne (Nb$_{\mathrm{CTEP}}$)	-0.90228	2.47968	1.75971	4.49086
Ne (Nb$_{\mathrm{CTEP}}$)	-2.48697	3.93674	-1.57260	4.22084	Ar (Y$_{\mathrm{CTEP}}$)	-1.33275	0.00453	-1.31279	2.06028
Ne (Nb$_{\mathrm{CTEP}}$)	-2.48697	1.81041	1.03263	4.27600	Ne (Nb$_{\mathrm{CTEP}}$)	0.90228	-2.47968	-1.75971	3.90225
Mg (Ca$_{\mathrm{CTEP}}$)	0.00000	-1.28747	1.30261	1.17326	Kr (Ti$_{\mathrm{CTEP}}$)	1.61066	2.41149	-1.72233	3.38610
Ne (Nb$_{\mathrm{CTEP}}$)	-2.48697	-1.81041	-1.57260	3.73580	Ar (Y$_{\mathrm{CTEP}}$)	3.83286	0.00453	-1.31279	2.22985
Ne (Nb$_{\mathrm{CTEP}}$)	2.48697	-1.81041	-1.03263	3.73580	Kr (Ti$_{\mathrm{CTEP}}$)	3.55495	-2.41149	1.72233	3.77170
Ne (Nb$_{\mathrm{CTEP}}$)	2.48697	1.81041	1.57260	3.94399	Ar (Y$_{\mathrm{CTEP}}$)	3.88644	0.00453	4.22981	2.46989
Ne (Nb$_{\mathrm{CTEP}}$)	2.48697	3.93674	-1.03263	4.22084	Kr (Ti$_{\mathrm{CTEP}}$)	1.66425	2.41149	3.82027	3.68924
Mg (Ca$_{\mathrm{CTEP}}$)	0.00000	4.45969	1.30261	1.72171	Ne (Nb$_{\mathrm{CTEP}}$)	4.26333	2.47968	1.75971	4.13169
q	1.49015	2.49607	-5.13821	-0.39915	q	-3.30873	-3.51108	2.00949	-0.39108
q	3.87253	0.79738	-4.54919	-0.58187	q	-2.74258	-1.35960	3.08018	-1.24958
q	3.63026	2.07620	-1.94396	-1.54156	q	-2.14674	-1.27629	0.48658	-0.54457
q	3.63026	3.67096	-4.54919	-0.86085	q	-0.72389	-3.77918	0.72734	-0.14259
q	1.49015	-3.25108	-5.13821	-0.05510	q	-0.69710	-3.54526	3.49864	-1.91622
q	-1.49015	-3.25108	-2.67747	-0.56643	q	1.85688	-3.51108	2.00949	-1.38773
q	-1.33773	0.55800	-4.69947	-0.66286	q	0.47439	-1.25002	5.04190	-0.81448
q	1.33773	-0.55800	-5.72145	-0.32421	q	1.88367	-3.81336	4.78079	-0.72444
q	-1.49015	-2.49607	-5.28270	-0.09452	q	-2.96529	1.27629	5.05602	-0.30415
q	1.49015	-2.49607	-2.53298	-0.15172	q	-2.36945	1.35960	2.46242	-0.51699
q	-3.63026	2.07620	-5.87172	-0.49436	q	-0.36723	1.25002	6.04330	-0.05648
q	-3.87253	0.79738	-3.26650	-0.12159	q	-2.09315	-1.27629	6.02918	-0.41758
q	-1.49015	3.25108	-5.28270	-1.28056	q	-1.83009	3.81336	0.76181	-0.87732
q	-3.63026	3.67096	-3.26650	-1.91014	q	0.75068	3.54526	2.04396	-1.88424
q	1.33773	6.30516	-3.11622	-0.01792	q	-1.80330	3.51108	3.53311	-0.98499
q	-1.33773	6.30516	-4.69947	-0.10080	q	-2.79616	-1.35960	-2.46242	-0.10020
q	1.33773	5.18915	-5.72145	-0.08290	q	-3.01887	1.27629	-0.48658	-0.48017
q	-1.33773	5.18915	-2.09424	-0.69211	q	-2.42303	1.35960	-3.08018	-0.13736
q	-3.63026	2.07620	-0.66127	-1.54156	q	-0.22060	-1.35633	-3.13254	-0.83523
q	-3.87253	4.94978	-0.66127	-0.75621	q	0.16702	1.35633	-2.41006	-0.12777
q	-1.49015	2.49607	2.53298	-0.39915	q	1.80330	-3.51108	-3.53311	-0.47671
q	-3.87253	0.79738	1.94396	-0.58187	q	2.36945	-1.35960	-2.46242	-0.23435
q	-3.63026	3.67096	1.94396	-0.86085	q	1.83009	-3.81336	-0.76181	-0.60650
q	1.49015	-3.25108	0.07225	-0.56643	q	-0.75068	-3.54526	-2.04396	-0.83600
q	-1.49015	-3.25108	2.53298	-0.05510	q	0.69710	3.54526	-3.49864	-0.20173
q	1.33773	0.55800	2.09424	-0.66286	q	0.72389	3.77918	-0.72734	-0.38496
q	-1.49015	-2.49607	-0.07225	-0.15172	q	2.74258	1.35960	-3.08018	-0.86567
q	-1.33773	-0.55800	3.11622	-0.32421	q	3.30873	3.51108	-2.00949	-0.65247
q	1.49015	-2.49607	2.67747	-0.09452	q	4.94501	-1.35633	-3.13254	-0.12488
q	-3.63026	-3.67096	-0.66127	-0.26930	q	5.33263	1.35633	-2.41006	-0.23004
q	-3.63026	-2.07620	-3.26650	-0.59755	q	4.74480	1.25002	0.50070	-0.47467
q	-3.87253	-0.79738	-0.66127	-0.50992	q	5.58642	-1.25002	-0.50070	-0.35095
q	3.63026	-3.67096	-1.94396	-0.26930	q	4.99859	-1.35633	2.41006	-0.56533
q	3.63026	-2.07620	0.66127	-0.59755	q	4.44172	-3.77918	0.72734	-0.60249
q	3.87253	-0.79738	-1.94396	-0.50992	q	4.46851	-3.54526	3.49864	-0.79316
q	3.87253	0.79738	0.66127	-0.12159	q	2.20032	1.27629	5.05602	-0.36145
q	3.63026	2.07620	3.26650	-0.49436	q	5.38621	1.35633	3.13254	-1.54559
q	3.63026	3.67096	0.66127	-1.91014	q	4.79838	1.25002	6.04330	-0.39291
q	1.49015	3.25108	2.67747	-1.28056	q	5.64000	-1.25002	5.04190	-0.35864
q	1.33773	5.18915	-0.51099	-0.69211	q	3.07245	-1.27629	6.02918	-0.01313
q	3.87253	4.94978	-1.94396	-0.75621	q	0.77747	3.77918	4.81526	-0.54599
q	1.33773	6.30516	2.09424	-0.10080	q	3.36231	3.51108	3.53311	-1.81855
q	-1.33773	6.30516	0.51099	-0.01792	q	3.33552	3.81336	0.76181	-0.24423
q	-1.33773	5.18915	3.11622	-0.08290	q	5.91629	3.54526	2.04396	-0.75093
